# Unilateral Ureteral Obstruction as a Model to Investigate Fibrosis-Attenuating Treatments

**DOI:** 10.3390/biom9040141

**Published:** 2019-04-08

**Authors:** Elena Martínez-Klimova, Omar Emiliano Aparicio-Trejo, Edilia Tapia, José Pedraza-Chaverri

**Affiliations:** 1 Department of Biology, Faculty of Chemistry, National Autonomous University of Mexico (UNAM), Mexico City 04510, Mexico; emilianoaparicio91@gmail.com; 2 Department of Nephrology and Laboratory of Renal Pathophysiology, National Institute of Cardiology “Ignacio Chávez”, Mexico City 14080, Mexico; ediliatapia@hotmail.com

**Keywords:** unilateral ureteral obstruction, UUO, fibrosis, fibrosis-attenuating treatment

## Abstract

Renal fibrosis is the common pathway for most forms of progressive renal disease. The Unilateral Ureteral Obstruction (UUO) model is used to cause renal fibrosis, where the primary feature of UUO is tubular injury as a result of obstructed urine flow. Furthermore, experimental UUO in rodents is believed to mimic human chronic obstructive nephropathy in an accelerated manner. Renal fibrosis is the common pathway for most forms of progressive renal disease. Removing the obstruction may not be sufficient to reverse fibrosis, so an accompanying treatment may be of benefit. In this review, we have done a revision on treatments shown to ameliorate fibrosis in the context of the UUO experimental model. The treatments inhibit the production of fibrotic and inflammatory proteins such as Transforming Growth Factor β1 (TGF-β_1_), Tumor Necrosis Factor α (TNF-α), collagen and fibronectin, Heat Shock Protein 47 (HSP47), suppress the proliferation of fibroblasts, prevent epithelial-to-mesenchymal transition, reduce oxidative stress, inhibit the action of the Nuclear Factor κB (NF-κB), reduce the phosphorylation of mothers against decapentaplegic homolog (SMAD) family members 2 and 3 (Smad2/3) or Mitogen-Activated Protein Kinases (MAPKs), inhibit the activation of the renin-angiotensin system. Summaries of the UUO experimental methods and alterations observed in the UUO experiments are included.

## 1. Introduction

Obstructive nephropathy is a common clinical case [1] that can have irreversible long-term consequences: it can cause renal failure or interfere with kidney maturation [2]. Obstructive nephropathy may lead to Acute Kidney Injury (AKI) or Chronic Kidney Disease (CKD) [3,4,5]. CKD involves the progressive decrease in renal function and the alteration of renal structure lasting more than three months [6]. CKD is characterized by the loss of epithelial renal cells and their replacement with extracellular matrix (ECM) in the glomeruli and tubulointerstitium [5,6]. Patients with CKD are usually diagnosed after ECM accumulation has initiated [7] and show evidence of the effect of Reactive Oxygen Species (ROS)-induced damage in proteins, lipids and DNA [8]. In infants, the causes for obstructive nephropathy may be congenital [2,3] and may cause of end-stage renal disease [9].

### 1.2. Experimental Procedure of UUO

The Unilateral Ureteral Obstruction (UUO) is a model widely used to study obstructive nephropathy. The experimental procedure involves ligation of the ureter, usually with a silk thread, most commonly of the left one. The kidney of the ligated ureter is termed the Obstructed Kidney (abbreviated hereinafter as “OK”) and the contralateral second kidney is termed the Non-Obstructed Kidney (“NOK”). Table 1 includes a summary of the technical details of UUO experiments that are included in this review; in particular, the experimental animal used, the form of supplementation of the tested treatment, the type of anesthesia used and the details of ureteric ligation, as mentioned in each work. Male animals are preferred because female reproductive organs complicate the surgical procedure. The recommended anesthesia to reduce mortality is isoflurane/oxygen [2]. It is also necessary to maintain the animal on a surface heated to body temperature and use a good quality binocular microscope [2]. 

### 1.3. Events in the Unilateral Ureteral Obstruction (UUO) Model

**Figure 1:**
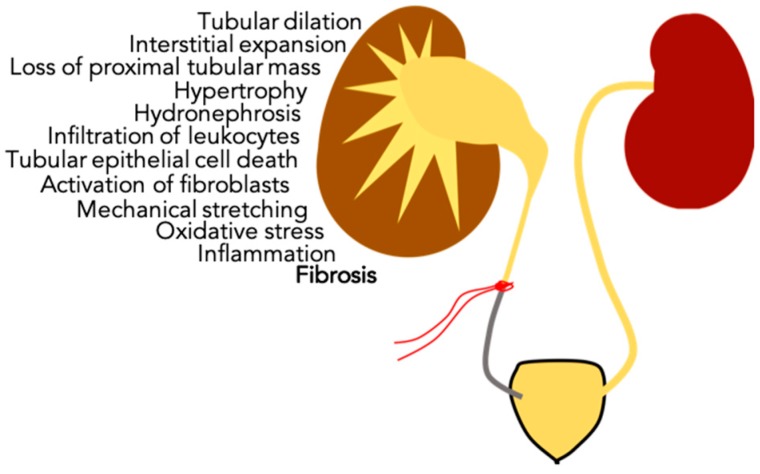
Summarized events that occur in the Unilateral Ureteral Obstruction-Obstructed Kidney (UUO-OK). The Unilateral Ureteral Obstruction (UUO) procedure typically consists of ligating one ureter with silk thread. Several variations on the technique have been reported and are listed in Table 1.

The UUO-obstructed kidney (UUO-OK) is characterized by tubular dilation, interstitial expansion, loss of proximal tubular mass, hypertrophy, hydronephrosis, infiltration of leukocytes, tubular epithelial cell death and presence of fibroblasts. These alterations are a result of molecular processes such as hemodynamic change by mechanical stretching, epithelial tubular cell apoptosis, oxidative stress and inflammation; which altogether lead to progressive renal tubulointerstitial fibrosis [2,4,10,11,12]. A summary of the events in UUO is shown in Figure 1. 

#### 1.3.1. Mechanical Stretching and RAS Activation

In the UUO model, the stationary urine flow causes the rise in the hydrostatic pressure, which initially dilates the collecting ducts. Then this pressure is transmitted back to the distal and proximal tubules. The rise in proximal tubule pressure has two effects: the decrease in the glomerular filtration rate (GFR) and mechanical stretching damage in tubular epithelial cells. Although at the earliest stage of UUO there is an increase in Renal Blood Flow (RBF) that lasts 1–2 h but the permanence of the obstruction results in the progressive decrease in RBF and increase in renal vasoconstriction, which may lead to inadequate blood supply (ischemia). The activation of both, the renin-angiotensin-aldosterone system (RAS) and endothelin-1 (ET-1) also contribute to the renal vasoconstriction and the posterior processes of tubular epithelial cell apoptosis, oxidative stress inflammatory response and fibrosis. 

#### 1.3.2. Tubular Epithelial Cell Apoptosis

The initial mechanical stretching causes damage and the apoptosis of the tubular epithelial cells [3]. Other causes for the apoptosis of renal tubular cells are oxidative stress [8,13] and the secretion of pro-apoptotic soluble factors [5]. Tubular cell apoptosis has been observed as early as 3UUO (at 1UUO tubular cell apoptosis was not observed [14]) and the severity increases with UUO-time [14]. Hereinafter, a number placed before “UUO” will indicate the number of days of UUO; for example, “3UUO” means 3 days of UUO.

#### 1.3.3. Oxidative Stress

In the UUO model, oxidative stress contributes to renal damage progression since the early stages. ROS levels are increased after UUO, as well as the lipid peroxidation marker malondialdehyde (MDA), superoxide anion production and NAD(P)H oxidase (Nox) subunits protein levels [15]. Additionally, the antioxidant enzymatic system is decreased, the superoxide dismutase and catalase levels are decreased in the UUO group at 14UUO [13,15]. The levels of Nuclear Factor (Erythroid-derived 2)-Like 2 (Nrf2)—a transcription factor that translocates to the nucleus and initiates transcription of antioxidant response elements—are also reduced. Under oxidative stress, Nrf2 translocates to the nucleus and initiates transcription of antioxidant enzymes such as: glutathione peroxidase, NAD(P)H:quinone oxidoreductase 1 (NQO1), catalase, heme oxygenase 1 (HO-1), thioredoxin reductase and the glutamate-cysteine ligase catalytic subunit [8]. However, in the UUO model, RAS activation and the increase in Nuclear Factor κB (NF-κB) could downregulate Nrf2 expression in rat kidneys [36]. RAS activation is probably the inducer for ROS production increase by the Nox [37] and the suppression of Nrf2. Additionally, RAS activation stimulates the production of factors that trigger leukocyte migration and the inflammatory process, namely: Transforming Growth Factor β1 (TGF-β_1_), Tumor Necrosis Factor α (TNF-α), Monocyte Chemoattractant Protein 1 (MCP-1), Vascular Cell Adhesion Molecule 1 (VCAM-1) and NF-κB. 

#### 1.3.4. Inflammation

As a consequence of oxidative stress, RAS activation and stimulation by cytokines, the NF-κB transcription factor translocates to the nucleus, where NF-κB initiates the transcription of target genes that cause inflammation and fibrosis [20]. There is a reinforcing loop between NF-κB and RAS; and between NF-κB and TNF-α [13]. RAS genes are regulated by the Wnt/β-catenin pathway [25]. RAS provokes fibrosis by triggering TGF-β/Smad and Wnt/β-catenin signaling. It is a vicious circle because fibrosis then further activates RAS and there is further activation of TGF-β/Smad and Wnt/β-catenin signaling pathways. The target proteins of the Wnt/β-catenin signaling pathway are: Snail1, Twist, Matrix Metalloproteinase 7 (MMP7), Plasminogen Activator Inhibitor 1 (PAI-1) and Fibroblast Specific Protein 1 (FSP-1 a.k.a. S100A4) [13]. For instance, Snail1 is a transcription factor that is a master regulator of Epithelial-to-Mesenchymal Transition (EMT) [38]. Snail1 has been observed to increase in the UUO-OKs [38].

#### 1.3.5. Leukocyte Migration

When injured, the parenchymal, endothelial and tubular epithelial cells of the kidneys secrete exosomes, chemokines and cytokines [38]. In response, leukocytes (mainly macrophages, T-lymphocytes and neutrophils), are recruited to the tubulointerstitium [5]. Such migration starts from day one of UUO and lasts throughout UUO [5]. In the UUO model, macrophages are accumulated in the renal cortex and promote inflammation [10]. The area that attracts the infiltrating macrophages is marked by the presence of adhesion molecules such as selectins [3]. By the upregulation of MCP-1 expression, TNF-α recruits monocytes and macrophages towards the tubulointerstitium of the UUO-kidneys [9]. Monocytes and macrophages, in turn, produce TNF-α and TGF-β_1_ [9]. TNF-α also stimulates inflammation [9]. 

#### 1.3.6. Fibroblast Activation

Renal interstitial fibroblasts and myofibroblasts are mostly responsible to produce excessive ECM in the fibrotic kidney [39]. The more severe the degree of fibrosis, the more myofibroblasts are in the kidney [5]. Myofibroblasts are long-term activated fibroblasts that express alpha Smooth Muscle Actin (α-SMA) [2]. Interstitial fibroblasts transform into myofibroblasts [2]. According to LeBleu et al. [40] 50% of myofibroblasts in kidney fibrosis arise from local resident fibroblasts, 35% arise from the bone marrow, 10% arise by endothelial-to-mesenchymal transition and 5% by epithelial-to-mesenchymal transition. Fibroblasts acquiring a myofibroblastic phenotype are a crucial event for expanding the ECM [41]. The most abundant isoform of the TGF-β family, TGF-β_1_, is significantly increased in the UUO-OKs [9]. TGF-β_1_ is the main factor that activates fibroblasts to produce ECM and myofibroblasts are the principal cells that produce fibrotic ECM [5,42]. TGF-β_1_ can also induce EMT via both: Smad-dependent and Smad-independent signaling pathways [38]. The EMT process allows tubular epithelial cells to acquire the phenotype of a mesenchymal cell, enhanced migratory capacity, elevated resistance to apoptosis and increased production of ECM [2,38,43]. 

#### 1.3.7. Fibrosis

Renal fibrosis is a final common pathway in the progression from CKD towards end-stage renal disease [38] and can be a potentially lethal disease [44]. In addition, the deterioration of kidney-function correlates strongly with renal fibrosis [27], so inhibiting renal fibrosis could delay or prevent CKD [38]. The terms “fibrosis,” “sclerosis” and “scarring” are used as synonyms [2]. The accumulation of ECM is what leads to fibrosis and tissue scarring [39]. Injured tubular cells contribute to a thickened tubular basement membrane composed mainly of collagen type IV but fibroblasts are the main sources of tubulointerstitial ECM production [5]. In addition to collagen types I, III and IV, the ECM is composed of sulfated and non-sulfated glycosaminoglycans, such as biglycan and decorin and fibronectin [41]. Tubular atrophy appears to be more closely associated with renal malfunction and interstitial fibrosis than the injury of the glomeruli in the UUO model [1]. For instance, at day 21 of UUO, no significant pathological changes were observed in kidney glomeruli [39]. Moreover, there is a strong correlation between progressive tubular epithelial cell death with the progression of fibrosis [2] and more severe renal tubulointerstitial fibrosis is observed in the renal cortex [10]. In rodents, the fibrosis can be more severe in neonates than in adult animals [2]. Experimental UUO in rodents is believed to mimic human chronic obstructive nephropathy in an accelerated manner. In short, the UUO model causes renal fibrosis and tubular injury as a result of the obstructed urine flow [1]. The advantage of the UUO model is that it is possible to remove the obstruction and then study the subsequent events [2,5]. 

### 1.4. Justification

Therapeutic strategies for renal fibrosis caused by ureteral obstruction are currently under study [27], because removing the ureteral obstruction may not be sufficient to stop kidney damage progression: Chevalier et al. [45] and Chaabane et al. [11] reported that the release of the obstruction did not reverse the reduction in the number of nephrons and did not improve tubular cell proliferation, although the glomerular filtration rate returned to normal [45]. Ito et al. [46] found that the levels of TGF-β_1_ continued to rise, even one month after release of the obstruction. So, a pharmacological treatment may prove beneficial in delaying or preventing the loss of kidney function [46]. 

In this review, we have revised the subject of the treatments demonstrated to ameliorate fibrosis in the context of the UUO experimental model. We have also compiled information on the technical details of the UUO experiments and we have listed (1) the histology techniques used to visualize damage in the kidney tissues, (2) the antibodies used for immunohistochemistry and immunofluorescence, (3) the primers used to quantify gene expression by reverse-transcription quantitative polymerase chain reaction (RT-qPCR) and (4) the antibodies used to quantify protein levels by Western Blot. We summarize the alterations that have been observed in the UUO-experiments and how they compare after treatment administration. 

### 1.5. Histology and Immunohistochemistry

Histological staining techniques have shown that, in the UUO-OKs, there is severe structural damage, a decreased number of tubules, tubular atrophy, dilated tubules, cystic dilatation, infiltration of inflammatory cells, tubular epithelial cell apoptosis, tubulointerstitial fibrosis and accumulation of ECM [1,10,17,20,35]. The tubular lumen area has been measured to increase 5-fold in mice with UUO [32]. 

Periodic acid schiff (PAS) staining has been used to assess histological changes, especially glomerulosclerosis, glomerular volume, as well as the number of tubules and their degree of destruction [11,14]. PAS is helpful for staining basement membranes [41]. Masson’s trichrome staining is used to estimate the grade of interstitial fibrosis, since the collagen fibrils in the interstitial area are stained green or blue, depending on the dye used [47]. Masson’s staining is helpful to visualize both basement membranes and interstitial fibrosis [41]. With Masson’s staining, it has been observed that at 7UUO and 14UUO, the ECM accumulation thickens the interstitial space and the tubular basement membrane [5,34]. Sirius Red is used to stain collagen I, III and IV [2,26,32]. Sirius Red-positive areas in the UUO-OK increased progressively with UUO-time [26], being observable already at 7UUO [38]. Sirius Red staining also revealed collagen deposition around the glomeruli and in the tubulointerstitial compartments of UUO-OKs [32]. Interestingly, with Sirius Red, collagen deposition inside the glomeruli was not observed [32]. Immunohistochemical staining is performed to identify antibody-specific staining-positive areas of renal cortex sections. Immunohistochemistry has shown that deposition of collagen I, III and IV increased in the tubulointerstitium of the UUO-OKs [17,29,34]. TGF-β_1_ has been detected in the interstitial areas of the UUO-OKs [38], its intensity increasing with UUO-time. 

There are two types of macrophages: M1 (anti-ED3 or CD169) and M2 (anti-ED2 or CD163): M1 macrophages (the classically-activated macrophages that promote inflammation and apoptosis) become active as the initial line of defense and invade the renal cortex of the UUO-OK. Second, to counteract the increase in M1 macrophages, M2 macrophages (the alternatively-activated macrophages that promote cell survival and fibrogenesis) invade the inflammation site to prevent further inflammation. The increase in M2 macrophages produces an increment in the levels of TGF-β_1_, which results in the induction of fibrosis [5,10]. Significant correlations have been observed when comparing the fibrotic area and the collagen I-positive area with the number of M2 macrophages in the UUO-OKs [10]. Double immunohistochemical staining for macrophages and TGF-β_1_ showed the presence of giant multinucleated cells in renal cortex sections of the UUO-OK [10], possibly originating from the fusion of macrophages. CD3 was used as a marker of lymphocytes and was increased in the UUO-OK [48]. 

In immunohistochemical determinations, the staining-positive area obtained with each of the antibodies has generally been divided between the renal area and expressed as its percentage. The presence of myofibroblasts can be identified by looking at α-SMA, vimentin, fibronectin and collagen IV expression [1,42]. The most abundantly observed proteins in UUO-OKs are fibronectin and collagen I, which increase significantly at 7UUO [42]. Fibronectin deposition in the UUO-OKs was increased in the glomeruli and in the tubulointerstitial compartment, compared to Sham-kidneys. The immunohistochemically-observed increase in fibronectin and collagen I was already significant at 7UUO [42]. At 1UUO, swelling of the renal tubular epithelial cells was observed [14]. At 3UUO, inflammatory cell infiltration, tubular atrophy, tubule expansion and interstitial fibrosis were detected [14]. At 7UUO, interstitial fibrosis was already detected together with tubular atrophy, blood vessel collapse, inflammatory cell infiltration and heightened ECM production [14]. At 14UUO, fibrosis was very evident and more severe than at 7UUO, with the majority of tubules destroyed and with marked disorganization in the structure of proximal and distal tubules [14]. The interstitial area in UUO-OKs is greater than that in Sham-kidneys at 7UUO and 14UUO [14]. 

In the renal cortex sections of UUO-OKs, immunohistochemical-staining showed increased α-SMA–positive areas and progressively increase with UUO-time, compared to Sham-kidneys [26]. α-SMA–positive areas have been observed in UUO-OK cortex sections, but in a much larger proportion compared to the UUO-NOKs or Sham-kidneys [10]. Consider that α-SMA–positive areas are also present in the walls of blood vessels of Sham-kidney cortex sections [10]. But in Sham-kidneys, the α-SMA–positive area is limited to the vascular smooth muscle cells of blood vessel walls [19,32]. In UUO-OKs, vimentin- and α-SMA–positive areas have been found all over the tubulointerstitial space, especially surrounding the peritubular and periglomerular areas [19,42]. In some studies, the α-SMA–positive area was higher at 7UUO than at 14UUO compared to Sham [26]. Dual staining with α-SMA and Proliferating Cell Nuclear Antigen (PCNA) has been used to indicate the proliferation of fibroblasts [49]. 

EGF-like module-containing mucin-like hormone receptor-like (F4/80) is a marker of murine macrophages [24]. F4/80-positive macrophages increased in the tubulointerstitial areas of the UUO kidneys [8,29,32]. The protein FSP-1 (a.k.a. S100A4) is an early marker of the fibrotic activity of fibroblasts that have been derived from epithelial cells [35]. FSP-1 immunochemical staining has been detected in the UUO-OKs and increased progressively with UUO-time, specifically in the interstitial areas and in tubular epithelial cells but not in Sham-kidneys [35]. Cyclin-dependent kinase inhibitor 2A (P16^INK4a^) is involved in regulating the cell cycle. In the UUO-OKs, Smad2/3- and P16^INK4a^-positive staining have been found predominantly in the nuclei of tubular epithelial cells; above all, in atrophic and dilated tubules [35]. 

At 14UUO, FSP-1–positive fibroblasts have been detected in the tubulointerstitial area of UUO-OKs. Ki-67 is a marker of cell proliferation. Double-positive α-SMA and Ki-67 myofibroblasts increased at 14UUO in the OK’s tubulointerstitial area, compared to Sham-kidneys. Intercellular Adhesion Molecule 1 (ICAM-1) –positive areas have been observed in the interstitial areas of the UUO-OKs [29]. MCP-1–positive areas increased in the damaged dilated tubules and in the interstitial areas of the UUO-OKs [29,34], showing a stronger increase at 14UUO than at 7UUO [14]. On the other hand, E-cadherin is involved in cell adhesion and its reduction triggers loss of epithelial cell adhesion. E-cadherin levels decreased in the UUO-OKs [38], which indicates loss of tubular integrity [14,32]. The decrease in E-cadherin protein levels is seen already at 3UUO [7] and becomes stronger with UUO-time. Although surprisingly, E-cadherin mRNA levels have been found to increase in the first few days after UUO [7] but then decrease with UUO-time. The simultaneous increase in α-SMA and decrease in E-cadherin are an indication of epithelial cells undergoing EMT [38]. 

The transmembrane phosphoglycoprotein CD34 is a marker of vascular endothelial cells. It has been used to assess peritubular capillary changes, finding a reduction in peritubular capillary density at 3UUO that is more evident at 14UUO [14]. Hypoxia Inducible Factor 1α (HIF-1α) has been detected in tubular epithelial cells and fibrotic areas, the increase in HIF-1α protein levels being more pronounced at 14UUO. Vascular Endothelial Growth Factor (VEGF) has been detected in glomerular podocytes and tubular epithelial cells. VEGF protein levels decreased at 14UUO [14]. Table 2 includes a summary of the antibodies used for immunohistochemical staining in each of the studies included in this review.

### 1.6. RT-qPCR

mRNA levels of specific genes have been determined in certain studies. Table 2 shows a summary of the genes each investigation focused on. In the studies included in this review, the protein expression levels were quantified by Reverse-Transcription quantitative Polymerase Chain Reaction (RT-qPCR) and normalized with the level of transcription of the glyceraldehyde 3-phosphate dehydrogenase (GAPDH, G3PDH) gene or the actin gene. 

In the UUO-OKs, increased mRNA levels were observed of α-SMA, apoptosis-associated speck-like protein containing a CARD (ASC, a.k.a. TMS1 or PYCARD), collagen I, III and IV, fibronectin, P16^INK4a^, Nucleotide-binding Oligomerization domain-like Receptor containing Pyrin domain 3 (NLRP3), TGF-β_1_, TGF-β_1_ Receptor (TGF-β_1_R), TNF-α, Smad2, Smad3, p-Smad2, p-Smad3, pro-caspase-1, osteopontin (OPN), VCAM-1, in comparison to Sham-kidneys [19,26,32,38]. The increase in mRNA levels of α-SMA, fibronectin, collagen, TGF-β_1_ was much more pronounced at 14UUO than at 7UUO [1]. Expression of the pro-apoptotic genes Bax1 and Bim increased, while expression of the antiapoptotic regulator gene B-cell lymphoma 2 (Bcl-2) decreased [8]. The attenuated expression of BMP-7 leads to EMT [43].

### 1.7. Western Blotting

The protein expression levels are quantified by densitometry and normalized with the level of translation of the GAPDH gene (a.k.a. G3PDH) and therefore expressed as the relative abundance of a protein. The levels of phosphorylated proteins are also normalized but with the levels of the same non-phosphorylated protein and are also expressed as the relative abundance of the protein. 

UUO procedures are well-known for the consistently increased TGF-β_1_ mRNA and protein levels [4,17,32,35]. TGF-β_1_ protein levels have been shown to be either the same at 7UUO and 14UUO [50] or higher at 14UUO than at 7UUO [1]. α-SMA protein levels are increased in UUO-OKs compared to Sham-kidneys [17,38]. α-SMA protein levels have also been shown, either to be the same at 7UUO and 14UUO [50] or higher at 14UUO than at 7UUO [1]. Fibronectin protein levels increased in the UUO-OKs [32]. Fibronectin protein levels were higher at 14UUO than 7UUO [1,50]. Collagen I, III and IV protein levels increased in UUO-OKs [17,24]. Collagen I levels appeared higher at 7UUO than 14UUO [14,50]. Collagen IV was higher at 14UUO than at 7UUO [1]. PCNA protein levels are increased after UUO [15]. 

**Figure 2:**
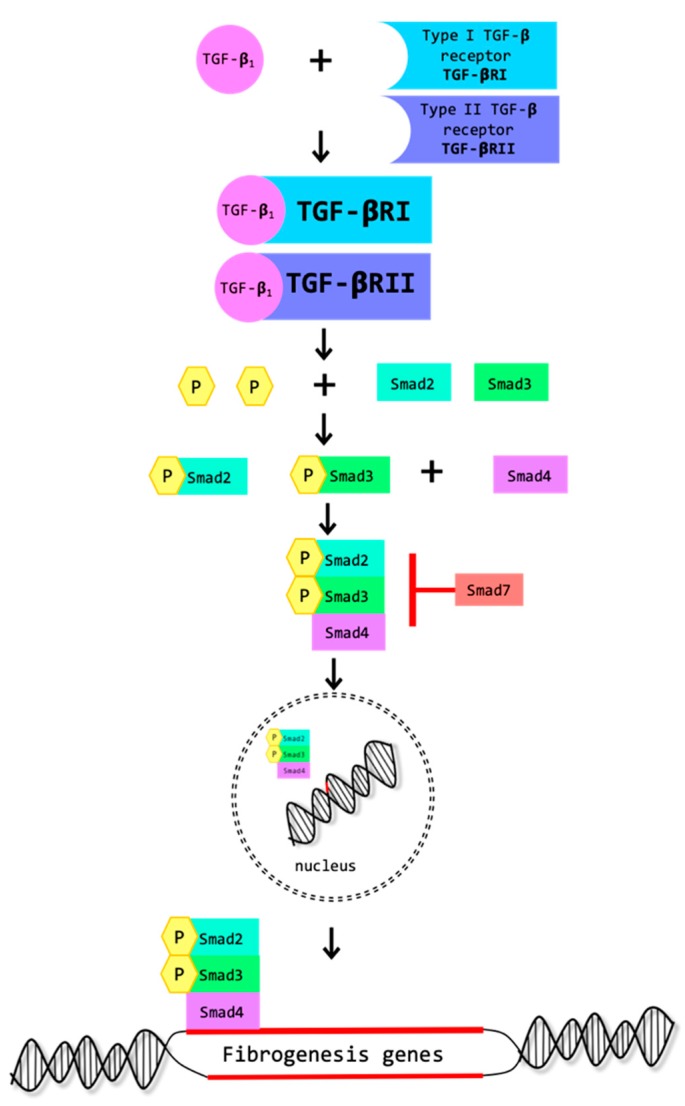
Smad-dependent onset of fibrosis. TGF-β_1_ binds to TGF-β Receptors type I and II (TGF-βRI and TGFβ-RII), involving Smad2/3, which must become phosphorylated in order to form a complex with Smad4. Smad7 has the capacity to repress the complex. The complex translocates to the nucleus, where it is required for the transcription of their target fibrogenesis genes.

TNF-α plays a role in renal inflammation. TNF-α protein levels increased in the UUO-OKs [32]. Smad2, Smad3 and Smad4, protein levels increased in the UUO-OKs [17,27]. The phosphorylated forms of Smad2 (p-Smad2) and Smad3 (p-Smad3) are the active forms that are involved in the onset of fibrosis and are increased in the UUO-OKs [29,38]. p-Smad2 and p-Smad3 are required for Smad-dependent EMT-onset caused by TGF-β1, as depicted in Figure 2. Smad1/5/8 protein levels decreased in the UUO-OKs compared to Sham-kidneys [35]. Smad7 protein levels also became depleted after UUO [29]. 

In the UUO-OK, cell cycle arrest has been reported, possibly caused by TGF-β and Smad2/3 increase, which induce the expression of the cyclin-dependent kinase inhibitor P16^INK4a^ by keratinocytes. In line with this, increased protein levels of P16^INK4a^ have been found in the UUO-OKs [35]. VCAM-1 protein levels were increased in the UUO-OKs [32]. Toll-like receptor 4 (TLR4) participates in the regulation of the inflammatory and fibrotic response by interacting with NF-κB [20]. The UUO-OKs showed higher levels of TLR4. Matrix Metalloproteinases (MMPs) function in ECM remodeling. Tissue inhibitors of metalloproteinases (TIMPs) are specific inhibitors of MMPs. MMP-2 and TIMP-1 protein levels are higher in the UUO-OKs compared to the Sham-kidneys [17]. 

Kinases IκKα, p-IκKα, IκBα, p-IκBα are also involved in the signaling pathway of the transcription factor NF-κB, which forms a complex with DNA. The UUO-OKs showed higher levels of IκKα, p-IκKα and p-IκBα; but lower levels of IκBα [17]. When IκBα phosphorylation (p-IκBα) is decreased, the nuclear accumulation of NF-κB is decreased. The Mitogen-Activated Protein Kinases (MAPKs) signaling pathways regulate apoptosis and inflammation. In the UUO-OKs, protein levels of c-Jun N-terminal kinase (JNK) MAPK, phosphorylated-JNK MAPK, extracellular signal-regulated kinase 1/2 (ERK1/2) MAPK, phosphorylated-ERK1/2 MAPK, p38 MAPK and phosphorylated-p38 MAPK are increased at 14UUO [1]. The phosphorylated proteins are the active forms. p-ERK1/2 is required for Smad-independent EMT-onset caused by TGF-β_1_. In Reference [7], the phosphorylation of JNK and ERK was increased in the UUO-OKs but the phosphorylation of p38 MAPK did not increase. ERK and JNK signaling also modulate the expression of the Heat Shock Protein 47 (HSP47) that is expressed by interstitial myofibroblasts, tubular epithelial cells, periglomerular and peritubular interstitium of the UUO-OKs. HSP47 has been scarcely observed in the UUO-NOKs. HSP47 is involved in the development of fibrosis, being a collagen-specific chaperone. HSP47 protein expression was increased in the UUO-OKs [34]. Compared with Sham-kidneys, other protein levels that also increased progressively in the UUO-OKs were: the cleaved caspase-3 [53], which participates in apoptosis; the cleaved caspase-1 and pro-caspase-1 [48]; angiotensin II Type 1 Receptor (AT1R) [8]; NLRP3 and ASC [48].

Klotho is a protein bound to the membrane of kidney distal tubules that has anti-fibrotic effects in the kidney [20]. During UUO, Klotho protein levels are reduced [31]. Acetylated-α-tubulin, α-tubulin and vimentin protein levels have been determined to evaluate altered microtubules [53]. Vimentin and acetylated-α-tubulin levels were increased in UUO-OKs, compared to Sham-kidneys [53]. In the OK, cytoplasmic Nrf2 protein levels were reported to either decrease [8] or remained the same as in Sham-kidneys [30]; while nuclear Nrf2 protein levels were reported to either remain the same [8] or increase [30]. Protein levels of the Nrf2-dependent antioxidant enzymes NQO1, HO-1, copper-dependent Superoxide Dismutase (CuSOD), manganese-dependent Superoxide Dismutase (MnSOD) and catalase, are decreased. 

Tumor suppressor ataxia telangiectasia mutated (ATM) is involved in the TGF-β_1_ pathway [55]; Phosphorylated-ATM (pATM) is the active form [55]. Nox promote the phosphorylation of ATM and p53 that is necessary for TGF-β_1_ induced fibrogenesis [55]. p22^Phox^ is a subunit of Nox that is elevated in UUO-OKs [55]. The Epidermal Growth Factor Receptor (EGFR) is another protein that is increased and phosphorylated in UUO [57]. Phosphorylation of EGFR also requires generation of ROS [57]. In addition, an increase in the phosphorylated-active form of Src was observed in the UUO-OKs [59]. Src is a tyrosine kinase that induces the phosphorylation of EGFR [59]. 

Protein levels of Wnt4, MCP-1, TNF-α, Interleukin 6 (IL-6), Interleukin 1 Beta (IL-1β), NF-κB, TLR4, α-SMA, fibronectin and TGF-β_1_ have also been determined using commercial assay kits [4,20,29,30,53], confirming that MCP-1 and TGF-β_1_ protein levels are increased in the tissue of the UUO-OKs [29]. Table 2 contains a summary of the proteins that were determined by Western Blot in each of the studies included in this review.

### 1.8. Fibrosis-Attenuating Treatments for the UUO-OKs 

Table 3 includes a summary of the treatments that ameliorated renal fibrosis that have been included in the present review, including information regarding the dose supplied, the time and duration of supplementation, as well as the total duration of the UUO experiment. 

**Figure 3:**
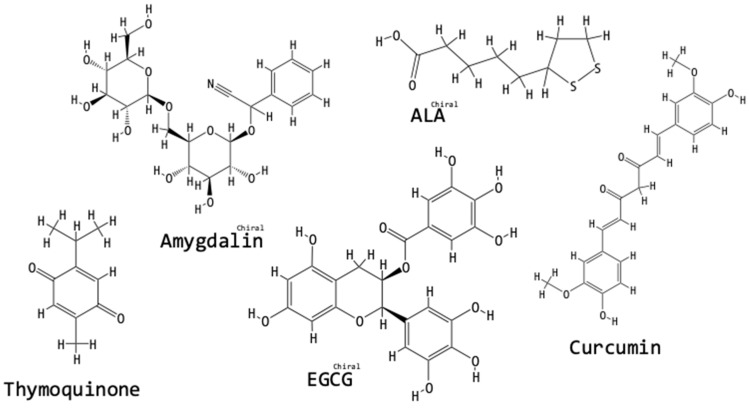
Chemical structures of vitamin and antioxidant compounds tested for fibrosis-ameliorating activity in the context of the UUO model. The structures were obtained from the PubChem database [60]: the “PubChem CID” for each compound has been listed in the text next to the compound’s name.

**Figure 4:**
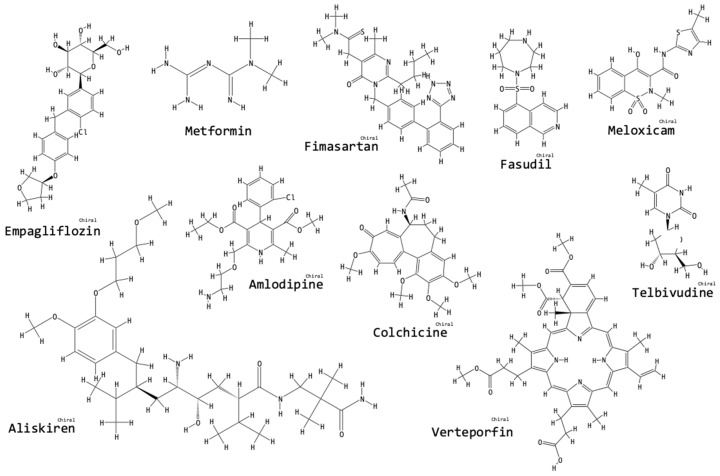
Chemical structures of pharmaceutical compounds tested for fibrosis-ameliorating activity in the context of the UUO model. The structures were obtained from the PubChem database [60]: the “PubChem CID” for each compound has been listed in the text next to the compound’s name.

**Figure 5:**
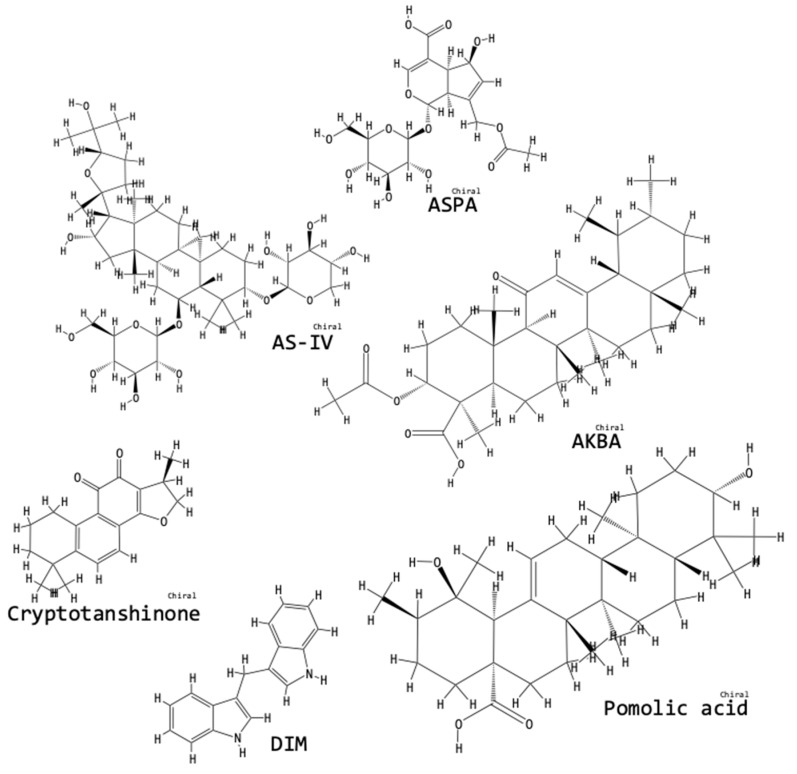
Chemical structures of plant-derived compounds tested for fibrosis-ameliorating activity in the context of the UUO model. The structures were obtained from the PubChem database [60]: the “PubChem CID” for each compound has been listed in the text next to the compound’s name.

**Figure 6:**
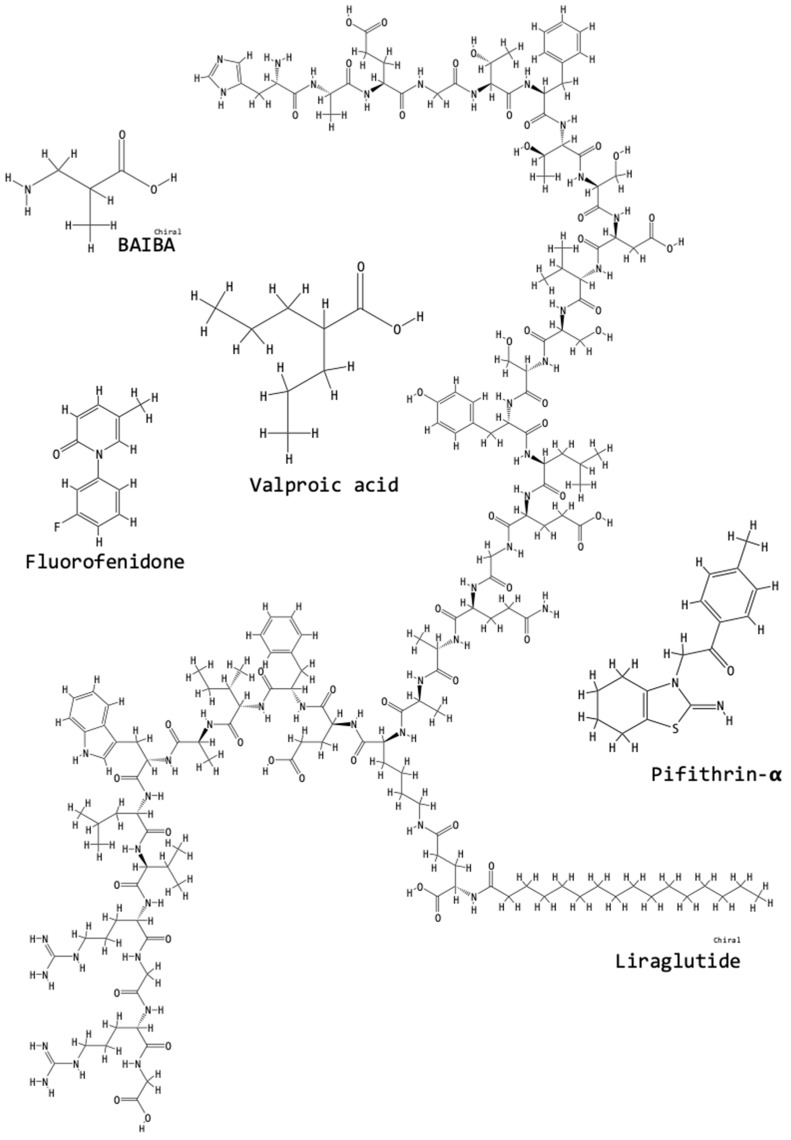
Chemical structures of purified or synthesized compounds and a selected recombinant protein tested for fibrosis-ameliorating activity in the context of the UUO model. The structures were obtained from the PubChem database [60]: the “PubChem CID” for each compound has been listed in the text next to the compound’s name.

We searched for the names of all the compounds mentioned in this review in the online “PubChem” Open Chemistry Database of the National Center for Biotechnology Information, U. S. National Library of Medicine, National Institute of Health [60]. Listed next each compound name, we have added the “PubChem CID” number and in Figure 3, Figure 4, Figure 5 and Figure 6, we have incorporated the structures of the compounds as obtained from the PubChem database [60]. 

#### 1.8.1. Vitamins

Amygdalin—also known as vitamin B17 (PubChem CID 34751)—is a cyanide-containing compound found naturally in the seeds of fruits of the Rosaceae family. Guo et al. [39] found that daily injections of amygdalin delayed renal injury even at day 21 of UUO. In comparison to non-treated UUO-OKs, amygdalin-treated UUO-OKs showed reduced histological lesions, reduced ECM accumulation and reduced TGF-β_1_ levels. The mode of action of amygdalin is proposed to occur by suppressing the proliferation of fibroblasts in the kidney [39]. A review on the therapeutic use of amygdalin can be found in Reference [61]. 

#### 1.8.2. Antioxidants

Alpha-lipoic acid or “ALA” (PubChem CID 864) is a cofactor of several mitochondrial dehydrogenases, which can also act as an antioxidant. Wongmekiat et al. [4] found that the ALA-treated UUO-OKs showed minimized kidney injury compared to ALA-untreated UUO-OKs. With ALA-treatment, TGF-β_1_ levels in the UUO-OK were reduced compared to the non-treated UUO-OKs. MDA levels were also reduced, while glutathione levels and total antioxidant capacity were increased. The damage-attenuating effect is believed to be linked to ALA’s oxidative stress reduction capabilities or possibly to ALA’s preventive increase in NF-κB protein levels [4]. 

Curcumin (PubChem CID 969516) is a compound isolated from the rhizome of the plant *Curcuma longa* (Zingiberaceae), known for its bifunctional antioxidant and anti-inflammatory properties. Zhou et al. [49] found that in the UUO-OKs, curcumin dramatically induced the expression of the Peroxisome Proliferator-Activated Receptors Υ (PPAR-Υ). In the UUO-OKs, curcumin-treatment reduced the deposition of collagen, decreased the levels of p-Smad2/3 and inhibited the proliferation of fibroblasts [49]. Kuwabara et al. [18] also found that curcumin reduced the influx of macrophages towards the interstitium and attenuated fibrosis in the UUO-OKs. Curcumin treatment inhibited NF-κB activity and reduced the mRNA levels of TGF-β_1_ [18]. Hashem et al. [22] found that curcumin-treated UUO-OKs had reduced levels of TNF-α, TNF Receptor 2 (TNFR2) and caspase-8, while increasing the mRNA levels of TNFR1-Associated Factor 2 (TRAF2) and Receptor Interacting Protein (RIP); thus, revealing the antiapoptotic role of curcumin. In another experiment where the UUO was reversed, Hammad and Lubad [21] found that curcumin-treated UUO-OKs were less inflamed, which possibly resulted in detaining early mediators and thus, affecting less the glomerular hemodynamics. TNF-α levels in serum were decreased but no significant protective benefits were found with curcumin-treatment on renal function parameters after release of the obstruction [21]. For further information on the use of curcumin to prevent or treat renal fibrosis, the review by Sun et al. [62] is suggested. 

Thymoquinone (PubChem CID 10281) is derived from the seeds of *Nigella sativa* (Ranunculaceae). Hosseinian et al. [13] found that the thymoquinone-treated UUO-group had a higher total renal thiol content, higher activities of superoxide dismutase and catalase, as well as reduced apoptosis, compared to the untreated UUO-group. Protein levels of TNF-α, angiotensin II and MCP-1 were decreased in the treated UUO-group. It is possible that the mechanism of action of thymoquinone is by inhibiting angiotensin II production and thus, preventing oxidative stress [13]. 

Epigallocatechin-3-gallate or “EGCG” (PubChem CID 65064), produced by Biopurify Phytochemicals Ltd. China, is the most abundant catechin polyphenol extracted from the green tea plant *Camellia sinensis* (Theaceae) and was tested by Wang et al. [30]. Activity levels of the Glutamic-Pyruvic Transaminase (GPT) and Lactate Dehydrogenase (LDH) were used as indicators of renal function, as well as levels of Blood Urea Nitrogen (BUN) and Serum Creatinine (SCr), which were partly restored by EGCG-treatment in the UUO-group. Protein levels of TNF-α, NF-κB, IL-6 and IL-1β in the UUO-OKs were reduced with EGCG treatment. Protein levels of IκBα were increased with EGCG treatment but levels of p-IκBα were decreased. EGCG increased total bilirubin production, which is catalyzed by HO-1. EGCG also increased HO-1 levels and the nuclear accumulation of Nrf2, while inhibiting the translocation to the nucleus of NF-κB, thus preventing the formation of the DNA–NF-κB complex [30]. 

Figure 3 shows the structures of the vitamin and antioxidant compounds tested for fibrosis-ameliorating activity in the context of the UUO model.

#### 1.8.3. Pharmaceuticals

Aliskiren (PubChem CID 5493444) is a pharmaceutical drug produced by the company Novartis. Aliskiren blocks RAS and is a direct renin inhibitor, which reduces the production of angiotensin II. Wang et al. [28] found that inhibition of RAS prevented reduced expression of aquaporin AQP2 in the UUO-OK. Aliskiren treatment also decreased the inflammation in the UUO-OK, as well as the mRNA levels of TGF-β_1_ and TNF-α. Sakuraya et al. [19] studied the synergistic effect of mizoribine and aliskiren, finding that the combined therapy was more efficient than aliskiren alone because the number of CD68- and α-SMA–positive cells, tubular dilation, interstitial volume, were further decreased, as were TGF-β_1_, α-SMA, OPN, MCP-1 and renin mRNA levels [28]. 

Amlodipine (PubChem CID 2162) is a calcium channel blocker prescribed for hypertension. Honma et al. [24] found that blood serum samples of amlodipine-treated UUO-animals had slightly lower levels of BUN compared to untreated animals. Histological evaluation revealed that amlodipine reduced tubular dilation caused by UUO as well as the amount of fibrosis in the kidney sections. Amlodipine-treated UUO-OKs had slightly reduced mRNA levels of TGF-β_1_, HSP47, collagen IV. Amlodipine did not reduce the levels of collagen I, collagen III, nor α-SMA. Amlodipine treatment reduced the phosphorylation level of JNK MAPK but not ERK MAPK nor p38 MAPK. Amlodipine did not reduce macrophage infiltration after UUO [24]. 

Colchicine (PubChem CID 6167) is an anticancer medication. Kim et al. [53] found that compared to untreated UUO-OKs, colchicine-treated UUO-OKs had fewer tubular apoptotic cells; reduced protein levels of acetylated-α-tubulin, TGF-β_1_, fibronectin, cleaved caspase-3, caspase-9 and ectodysplasin A (ED1). Mechanical stress induces the hyper-polymerization of microtubules. Colchicin inhibits the polymerization of tubulin, thus ameliorating fibrosis. Colchicin also suppresses TGF-β_1_ expression, possibly by reducing the infiltration of inflammatory cells [53]. 

Empagliflozin (PubChem CID 11949646) is an inhibitor of the sodium-glucose linked transporter-2, currently used as an oral antidiabetic drug. Abbas et al. [20] found that empagliflozin-treated UUO-OKs showed lower SCr and BUN levels; reduced protein levels of TGF-β_1_, NF-κB, TLR4, α-SMA, fibronectin and Connective Tissue Growth Factor (CTGF). Also, empagliflozin-treated UUO-OKs showed increased protein levels of Klotho. Maybe empagliflozin treatment inhibits the TLR4/NF-κB pathway, thus inhibiting fibrosis [20]. 

Fasudil (PubChem CID 3547) is produced by Mitsubishi Tanabe Pharma Corporation (Japan). Baba et al. [26] studied the renoprotective effect of fasudil on UUO-OKs, finding that fasudil ameliorated renal interstitial fibrosis. Fasudil is an inhibitor of ROCK: Rho is a small G-protein and ROCK is a Rho-associated protein kinase that operates as the downstream effector of Rho. ROCK inhibitors may suppress the migration of cells [26]. Rho/ROCK is a signaling pathway involved in Chronic Kidney Disease. Fasudil supplementation reduced the mRNA level of collagen, TGF-β_1_ and α-SMA in the treated UUO-OK compared to the non-treated UUO-OK. Fasudil treatment reduced the Sirius Red-positive areas, the collagen content, hydroxyproline content (which is a reflection of collagen content), α-SMA and fibronectin levels in the treated UUO-OKs compared to the non-treated UUO-OKs. Fasudil might have inhibited TGF-β_1_–Smad signaling and macrophage infiltration into the injured kidneys [26]. 

Fimasartan (PubChem CID 9870652) is an angiotensin II receptor blocker used for lowering blood pressure. Fimasartan was administered in the form of “fimasartan potassium trihydrate.” Kim et al. [8] found that compared to untreated UUO-OKs, fimasartan-treated UUO-OKs showed fewer F4/80-positive infiltrating macrophages; reduced α-SMA–positive areas, reduced hydroxyproline content; reduced protein levels of AT1R, Nox1, Nox2 and Nox4; increased mRNA levels of NQO1, HO-1, Glutathione S-transferases GSTa2 and GSTm3, Bcl-2; increased protein levels of Nrf2, NQO1, HO-1, CuSOD, MnSOD and catalase. Fimasartan acts on the renin-angiotensin system, lowering production of ROS [8]. 

Meloxicam (PubChem CID 54677470) is a cyclooxygenase COX-2 inhibitor. Compared to non-treated OKs, Honma et al. [23] showed that meloxicam ameliorated interstitial fibrosis in the UUO-OKs. UUO-animals treated with meloxicam showed reduced SCr and urinary glucose and a slight reduction in BUN levels. Collagen IV mRNA levels were reduced. Meloxicam-treated UUO-OKs showed reduced phosphorylation of ERK and JNK MAPKs. Meloxicam possibly inhibits the expression of HSP47 and collagen IV [23]. 

Metformin (PubChem CID 4091) is used to treat type-2 diabetes. Cavaglieri et al. [32] found that metformin treated UUO-OKs had reduced F4/80-positive cells compared to untreated UUO-OKs, indicating that there was reduced macrophage infiltration in the UUO-OKs. The protein and mRNA levels of TGF-β_1_, TNF-α, fibronectin, collagen I and III, α-SMA were lower in treated, than in untreated UUO-OKs. There was reduced accumulation of fibronectin and collagen. The levels of E-cadherin increased in the treated UUO-OKs. Metformin increased renal Adenosine Monophosphate-activated Kinase (AMPK) activity. Metformin attenuated renal fibrosis and inflammation in the UUO-OKs possibly by inhibiting TGF-β_1_ expression [32]. 

PR-619 (LifeSensors, Malvern, PA, USA) is a pan-DUB (deubiquitinating enzyme) inhibitor. Soji et al. [27] have found that PR-619 attenuated renal fibrosis in the UUO-OK. The mice were administered PR-619 immediately after UUO. The supplementation of PR-619 suppressed renal fibrosis in the UUO-OK. The beneficial effect of PR619 was attributed to the promotion of protein degradation by the ubiquitin-proteasome degradation system, specifically by disrupting the TGF-β_1_ signaling. With PR-619 treatment, Smad4 protein levels were reduced. In the absence of Smad4, the heteromeric Smad2-Smad3-Smad4 complex (Figure 2) is probably no longer formed and as a consequence, there is no longer transcription of the Smad complex-target genes and the tissue-damaging effects of TGF-β_1_ are dissipated. The findings of Soji et al. are in accordance with previous similar findings regarding the beneficial effects of the lack of Smad4 [27]. 

Telbivudine (PubChem 159269) is an antiviral used against hepatitis B. Chen and Li [17] found that telbivudine-treated UUO-OKs appeared less damaged in histological evaluations compared to untreated UUO-OKs. Telbivudine treatment lowered the α-SMA–positive area as well as the α-SMA protein levels. Telbivudine treatment also lowered TGF-β_1_, Smad2, Smad3, p-Smad2 and p-Smad3 mRNA and protein levels compared to untreated UUO-OKs. NF-κB, p-NF-κB, MMP-2 and TIMP-1 protein levels lowered with telbivudine treatment. TLR4, IKKα, p-IKKα, IκBα, p- IκBα are proteins involved in the NF-κB signaling pathway: with treatment, TLR4, IκKα, p-IκKα and p- IκBα protein levels were decreased; while IκBα increased compared to untreated-UUO-OKs. The protein levels of the proinflammatory cytokines TNF-α and IL-1β decreased with telbivudine treatment. Telbivudine treatment could be used to improve renal function in patients with preexisting renal disease and in patients taking nephrotoxic treatments [17]. 

Verteporfin (PubChem CID 5362420) is administered for eye disease [56]. Verteporfin powerfully inhibits the Yes-Associated Protein (YAP) [56]. The Transcriptional Coactivator with PDZ-binding motif (TAZ) (Hippo signaling pathway) is a protein that accumulates in the nucleus of tubulointerstitial cells in UUO-OKs, compared to UUO-NOKs [33]. Anorga et al. suggest that TAZ is a fibrotic effector that should be a target for fibrosis-attenuating treatments, because TAZ participates in TGF-β_1_ signaling and there appears to be cooperation between the Hippo and the TGF-β_1_ signaling pathways, leading to fibrogenesis [33]. YAP is related to TAZ [33,56]. YAP and TAZ bind to Smad transcription factors and possibly the YAP/TAZ pathway regulates TGF-β/Smad signaling in fibroblasts [56]. Szeto et al. found that verteporfin-treated UUO-OKs showed decreased nuclear protein levels of YAP and TAZ, as well as reduced nuclear accumulation of Smad2/3. Protein levels of α-SMA and collagen IV were also reduced with treatment, compared to non-treated UUO-OKs. Verteporfin attenuated renal fibrosis by blocking fibroblast activation induced by TGF-β_1_ [56]. Figure 4 shows the chemical structures of pharmaceutical compounds tested for fibrosis-ameliorating activity in the context of the UUO model.

#### 1.8.4. Plant-Derived Compounds

Acetyl-11-keto-β-boswellic acid or “AKBA” (PubChem CID 11168203) is a pentacyclic triterpenoid compound that can be extracted from the plant *Boswellia serrata* (Burseraceae). Liu et al. [31] found that, compared to untreated UUO-OKs, AKBA-treated UUO-OKs showed decreased SCr and BUN levels; reduced protein levels of α-SMA, TGF-β_1_, collagen I, collagen IV, Smad 2/3, TGF-β type I and II receptors (TGF-βRI and TGF-βRII), p-Smad2/3 and Smad4; while increasing protein levels of Klotho and Smad7. AKBA has an effect over the Klotho/TGF-β_1_/Smad pathway [31]. 

Astragaloside IV or “AS-IV” (PubChem CID 13943297) is a saponin, extracted from the plant *Astragalus membranaceus* (Fabaceae). Xu et al. [1] found that, compared to non-treated UUO-OKs, AS-IV treatment ameliorated renal tubulointerstitial fibrosis of the UUO-OKs by alleviating major tubular loss, reducing morphological and structural changes, lowering SCr and BUN levels. AS-IV treated UUO-OKs showed lower mRNA and protein levels of fibronectin, collagen IV, α-SMA and TGF-β_1_. Treatment with AS-IV lowered the phosphorylation levels of p38, ERK1/2 and JNK MAPKs in UUO-OKs, compared to the non-treated UUO-OKs. AS-IV treatment was reflected in decreased caspase-3 activation, possibly mediated by p-p38 MAPK and p-JNK MAPK. It is possible that AS-IV can suppress the activation of the MAPK signaling pathway, thus reducing apoptosis and delaying fibrosis [1]. In another study, Wang et al. [54] found that AS-IV downregulated the expression of proteins in the Wnt/β-catenin signaling pathway in UUO rats because β-catenin was inhibited by AS-IV [54]. 

Asperulosidic acid or “ASPA” (PubChem CID 11968867) is an iridoid glycoside found in the plant *Hedyotis diffusa* (Rubiaceae), used in Chinese traditional medicine. ASPA is present in the Rubiaceae. Lu et al. [16] found that ASPA treatment reduced levels of BUN, uric acid and urinary protein. The protective effect of ASPA in the OKs was attributed to: the reduced mRNA levels of NF-κB, α-SMA, collagen III, fibronectin, TGF-βRI and TGF-βRII, the induction of PPAR𝝲 mRNA levels; as well as reduced protein levels of Smad2, Smad3 and Smad4 [16]. 

3,3’-diindolylmethane or “DIM” (PubChem CID 3071) can be extracted from cruciferous vegetables. Xia et al. [42] found that treatment with DIM reduced interstitial injury, as observed in histology sections. Immunohistochemistry showed that DIM treatment reduced levels of α-SMA–, vimentin-, fibronectin- and collagen I-positive cells, while the levels of E-cadherin increased. DIM possibly prevented EMT [42]. 

“Applephenon” (Asahi Co., Tokyo, Japan) is a commercial product composed of apple polyphenols from *Malus pumila* cv. Fuji (Rosaceae). Lee et al. [9] found that in UUO-OKs, Applephenon-treatment attenuated tubular dilation and interstitial volume; and reduced the expression of ED1, MCP-1, TGF-β_1_ and α-SMA. It is possible that Applephenon ameliorated renal fibrosis due to its anti-inflammatory capabilities by decreasing the expression of MCP-1 or ED1 and thereby decreasing the infiltration of macrophages [9]. 

Cryptotanshinone (PubChem CID 160254) can be isolated from the Chinese herb *Salvia miltiorrizha* (Lamiaceae). Wang et al. [51] found that cryptotanshinone treatment improved tubular expansion, prevented tubular atrophy and reduced infiltrating inflammatory cells, SCr levels and interstitial collagen deposition. Treated UUO-OKs had lower protein levels of collagen I, fibronectin, α-SMA and p-Smad3. Treated UUO-OKs retained their E-cadherin levels. It is possible that cryptotanshinone blocks EMT, thus ameliorating renal fibrosis [51].

Pomolic acid (PubChem CID 382831) is a pentacyclic triterpene that can be isolated from *Euscaphis japonica* (Staphylaceae), an Asian plant. Park et al. [52] found that, compared to untreated UUO-OKs, pomolic acid-treated UUO-OKs showed reduced collagen deposition, reduced infiltration of inflammatory cells in the tubulointerstitium, less swollen epithelial cells, reduced tubular atrophy, decreased mesangial area, reduced levels of EMT; reduced protein levels of collagen I, fibronectin, plasminogen activator inhibitor 1 (PAI-1), p-Smad3 and of the phosphorylated Signal Transducer and Activator of Transcription 3 (p-STAT). Pomolic acid is thought to inhibit the Smad3-STAT3 signaling pathway [52]. 

Poricoic acids are tetracyclic triterpenoid compounds that can be isolated from the pine-root fungus *Poria cocos* (Poliporaceae) used in traditional medicine, especially in Asia. Three different poricoic acids: PZC, PZD and PZE, were tested by Wang et al. [25], finding that, compared to untreated UUO-OKs, poricoic acid-treated UUO-OKs had attenuated EMT levels, decreased infiltration of inflammatory cells; reduced protein levels of p-Smad3, α-SMA, collagen I, fibronectin, Wnt and β-catenin; reduced mRNA levels of α-SMA. Poricoic acids also inhibited Smad3 phosphorylation and repress the activation of RAS and the Wnt/β-catenin signaling pathways [25]. Figure 5 presents the chemical structures of plant-derived compounds tested for fibrosis-ameliorating activity in the context of the UUO model.

#### 1.8.5. Purified or Synthesized Chemical Compounds

β-Aminoisobutyric acid or “BAIBA” (PubChem 64956) is an amino acid catabolite that was tested by Wang et al. [15]. In the treated UUO-OKs, protein levels of angiotensin II, α-SMA, collagen I, fibronectin and PCNA were reduced. BAIBA’s fibrosis ameliorating affects are possibly due to the inhibition of the AngII/IL-17/ROS pathway [15]. 

Fluorofenidone (PubChem 11851183) was synthesized by Zheng et al. [48] and tested in UUO, finding that, compared to the untreated UUO-OKs, fluorofenidone-treated UUO-OKs showed reduced renal damage and collagen deposition, lower infiltration of macrophages, leukocytes and T-lymphocytes, reduced cleavage of the IL-1β protein; reduced protein levels of cleaved caspase-1. Fluorofenidone reduced the maturation of IL-1β, by cleavage of pro-IL-1β to IL-1β. Fluorofenidone also reduced the activation of the NLRP3 inflammasome and weakened the interactions between ASC and NLRP3 and ASC and pro-caspase-1 [48]. 

LJ-1888, with the chemical formula (2R,3R,4S)-2-[2-chloro-6-(3-iodobenzylamino)-9*H*-purine-9-yl]-tetrahydrothiophene-3,4-diol, is an antagonist of the renal A_3_ adenosine receptor (A_3_AR). The MAPK pathway is involved with A_3_AR. Lee et al. [7] found that treatment with LJ-1888 reduced the mRNA levels of α-SMA, collagen I, fibronectin; reduced the protein levels of fibronectin, collagen I, lysyl oxidase (LOx) (which induces the cross-linking of the ECM), p-JNK and p-ERK MAPKs. Importantly, the delayed administration (after UUO) of LJ-1888, administered since day 3 of UUO and until day 10, also inhibited tubulointerstitial fibrosis in the UUO-OKs after fibrosis had already initiated. It is possible that the accumulation of ECM was reduced because the expression of LOx was reduced [7]. 

Pifithrin-α (PubChem CID 4817) is an inhibitor of p53. Overstreet et al. [58] found that p53 is required for the expression of PAI-1. In UUO, the expression of p53 is induced by TGF-β_1_. Compared to non-treated UUO-OKs, pifithrin-α treated UUO-OKs showed attenuated-fibrosis, reduced accumulation and phosphorylation of p53, as well as reduced apoptosis [63]. 

Valproic acid or “VPA” (PubChem CID 3121) is a branched short-chain fatty acid that is thought to regulate cell differentiation and apoptosis by inactivating histone deacetylases. Nguyen-Thanh et al. [29] investigated the effect of valproic acid (VPA) on UUO-OKs. VPA increased histone H3 acetylation in both, Sham-kidneys and in UUO-OKs. Compared to non-treated UUO-OKs, VPA-treated UUO-OKs showed reduced structural and morphological injury, reduced myofibroblast proliferation, reduced myofibroblast infiltration, decreased Sirius Red-positive areas, reduced TGF-β_1_, α-SMA, fibronectin, ICAM-1, MCP-1 and vimentin protein levels. The phosphorylation levels of Smad2 and Smad3 were reduced with VPA treatment in UUO-OKs. Smad7 protein levels in the UUO-OKs were increased with VPA treatment. Immunohistochemical staining revealed less F4/80-positive macrophage infiltration and less MCP-1–positive areas in the treated UUO-OKs. VPA possibly regulates macrophage infiltration or proinflammatory cytokine expression, thus reducing UUO-induced renal inflammation. The key to VPA activity may lie in the increased histone H3 acetylation [29]. 

#### 1.8.6. Recombinant Polypeptides 

Liraglutide (PubChem CID 16134956) is a glucagon-like peptide-1 (GLP-1) analog that exerts a glucose regulatory function, thus it has been used for the treatment of type 2 diabetes. Li et al. [38] found that, compared to untreated UUO-OKs, liraglutide-treated UUO-OKs had: reduced collagen deposits, reduced Snail1-, TGF-β_1_– and TGF-β_1_R–positive areas, reduced mRNA levels of fibronectin, α-SMA, TGF-β_1_, TGF-β_1_R and collagen I, reduced protein levels of p-Smad3 and p-ERK1/2; whereas levels of E-cadherin were increased. Liraglutide is possibly preventing EMT in the UUO-OKs [38].

Recombinant human erythropoietin (rhEPO) treatment was investigated by Tasanarong et al. [35] in the UUO-OKs. rhEPO has been used clinically in CKD patients for the treatment of anemia. Compared to the non-treated UUO-OKs, rhEPO treatment resulted in reduced histological damage, reduced FSP-1–positive areas and protein levels, TGF-β_1_-positive areas and protein levels were decreased, Smad2/3-positive areas and protein levels were decreased, Smad3 mRNA levels were decreased, P16^INK4a^-positive areas were diminished, mRNA and protein levels of P16^INK4a^ were reduced. In contrast, with rhEPO treatment, BMP-7 positive staining did not decrease as much as in the UUO-OKs and neither did BMP-7 mRNA and protein levels, Smad1/5/8 protein levels were increased, Smad8 mRNA levels were increased. rhEPO could be involved in inhibiting arrest of the cell cycle and epithelial-to-mesenchymal transition. rhEPO may have adverse effects: it may cause thrombosis [35]. Figure 6 shows the chemical structures of the purified or synthesized compounds and a selected recombinant protein tested for fibrosis-ameliorating activity in the context of the UUO model. 

#### 1.8.7. Small Interfering RNAs

HSP47 small interfering RNA (HSP47 siRNA) was investigated by Xia et al. [34], who found that treated UUO-animals had reduced HSP47, MCP-1 protein levels, reduced expression of collagen I, III and IV, alleviated fibrosis and reduced the extent of interstitial nephritis. The histological changes of UUO were significantly reduced with treatment. The number of CD68-positive cells was also reduced. When coupling the kidney transfection treatment of HSP47 siRNA with cationized gelatin microspheres as carriers for gene delivery, there was reduced tubulointerstitial damage, reduced HSP47 protein levels and reduced collagen accumulation at 14UUO compared to treatment with uncoupled HSP47 siRNA, indicating that the fibrosis-inhibiting effect of the HSP47 siRNA was more prolonged when coupling it with the gelatin carrier, due to the short half-life of siRNA molecules. Xia et al. have indicated the possibility of the association between HSP47 and macrophage infiltration to the UUO-OKs mediated by collagen-induced production of MCP-1 [34]. 

#### 1.8.8. Stem Cells

Treatment with stem cells is promising for recovery of kidney injuries. Sun et al. [14] investigated the effect that transplanting human amniotic fluid-derived stem cells (hAFSCs) into renal tissues. hAFSCs-treated UUO-OKs showed reduced renal damage, as well as a less expanded interstitial area. The reduction of peritubular capillary density was reduced with treatment. VEGF, E-cadherin, protein levels increased in treated compared to untreated UUO-OKs. In treated UUO-OKs, TGF-β_1_, HIF-1α, collagen I and MCP-1 protein levels decreased. Tubular cells proliferated more with hAFSCs-treatment at 14UUO than without it. Apoptotic cells in renal tubules were decreased with hAFSCs-treatment [14]. The hAFSC treatment is the only treatment reported in this review where tubular epithelial cells proliferate, indicating that treatment with hAFSCs is interesting and promising and most definitely requires further research. 

## 2. Discussion

In the UUO model, there is an increase in SCr and BUN levels in the UUO-animals compared to Sham-operated animals, which is generally much more evident at 7UUO than at 14UUO [1]. This can be explained by taking into account that both Scr and BUN are more closely associated to the acute damage stage rather than to the chronic stage; although at 14UUO, BUN and SCr levels are still significantly higher than in the Sham group. Histological findings reveal that the Masson trichrome staining positive area is higher in the renal sections of UUO-OKs than in Sham-operated renal sections. The positive area is higher at day 14 than at day 7 in UUO-OKs indicating increased extracellular matrix deposition at 14UUO. Additionally, the positive area stained with Masson trichrome significantly increased in the sections of kidneys that have received a nephrotoxic treatment, for example with fluoride [10], compared to non-treated rats.

Cell apoptosis is involved in the injury process of multiple kidney diseases. In the UUO model, the initial injury leads to epithelial cell apoptosis. Hosseinian et al. [13] determined that UUO-animals have a significant increase in renal cell apoptosis compared to Sham-animals. According to Xu et al. [1] renal tubular epithelial cell apoptosis contributes to both, tubular atrophy and renal fibrosis. Additionally, the persistent inflammation also contributes to renal damage [16].

Interstitial inflammation has been detected as early as 3UUO [16]. However, at 7UUO is when there are major changes in renal function and inflammatory protein expression levels are also much higher. Inflammation can be quantified by measuring myeloperoxidase (MPO) activity, which was significantly greater in the UUO group than in the Sham group [30]. Inflammation can also be measured by quantifying the levels of inflammatory cytokines, such as TNF-α, IL-1β, IL-6 and IL-17. Cytokine levels increase with the progressive increase in UUO-time [13,15,16] and have been found in tubular epithelial cells [17]. IL-1β promotes the secretion of other cytokines such as TNF-α and IL-β itself [48]. However, IL-1β maturation is involved in caspase-1 cleavage and inflammasome activation [48], which further promotes epithelial cell death. 

The mitogen-activated protein kinase (MAPK) pathway operates as a signaling pathway to promote inflammation and cell apoptosis [1]. Phosphorylation levels of p38, JNK and ERK MAPKs were increased in UUO-OKs after UUO, seen at 7UUO and at 14UUO [1,24]. For Xu et al. [1], the inhibition of the activation of caspase-3 has an anti-apoptotic effect. Caspase-3 activation is mediated by mitogen-activated protein kinase effectors phospho-p38-MAPK and phospho-c-Jun-N-terminal kinase (JNK-MAPK). Therefore, downregulation of JNK-MAPK is possibly associated with the inhibition of the activation of caspase-3 and therefore, reduced apoptosis. 

The increase in inflammation additionally triggers the fibrotic process, TNF-α has been reported to stimulate the production of TGF-β_1_ by fibroblasts, derived from the activation of the NF-κB pathway, because the NF-κB pathway serves to recruit macrophages. MCP-1 is a monocyte chemoattractant protein [64] that was reported to increase in UUO-renal sections compared to Sham [13]. There appears to be a regulatory loop between MCP-1 and TGF-β_1_; where an increase in MCP-1 leads to an increase in TGF-β_1_ [65], which promotes the fibrotic process. Fibrosis has been observed as early as 3UUO [35]. Serious fibrosis has been observed at 14UUO [16]. Tasanarong et al. [35] proposed two stages in the development of fibrosis: in the early stage, stress signaling (TGF-β_1_, p-Smad2/3) induces cell cycle arrest. In the second phase, there is EMT. High levels of α-SMA indicate the presence of myofibroblasts [26]. Using hematoxylin and eosin staining, nuclei are dyed brown or purple, while the cytoplasm is pink. 14UUO-OKs generally show a much larger number of nuclei and empty spaces and a smaller amount of cytoplasm than UUO-NOKs or Sham-kidney sections, suggesting the proliferation of fibroblasts. 

As we previously discussed, renal fibrosis is a common pathway in the progression of CKD and its transition to end-stage renal disease [38,44]. Fibrosis correlates strongly with the deterioration of kidney-function [27], so inhibiting renal fibrosis or the process that triggers fibrosis is being widely investigated to prevent the progression of CKD [38]. Ucero et al. [5] have suggested that renal fibrosis could be prevented by inhibiting JNK-MAPK or by inhibiting p53. p53 is a protein that regulates the cell cycle, while JNK-MAPK responds to cytokines and plays a role in apoptosis. High expression of the developmental gene Wnt4 has been reported in the fibrotic kidney, possibly associated with repair regulation. As Wang et al. have suggested [54], Wnt4 may be involved in pericyte-to-myofibroblast transition. It is not well understood if downregulation of the expression of proteins in the Wnt/β-catenin signaling pathway may contribute to inhibit renal interstitial fibrosis, since conditional deletion of Wnt4 in interstitial cells did not reduce myofibroblast proliferation during fibrosis. Also, Wang et al. [54] indicated that there might be crosstalk between the Wnt/β-catenin, TGF-β/Smad and integrin/ILK pathways, possibly promoting EMT. 

**Figure 7:**
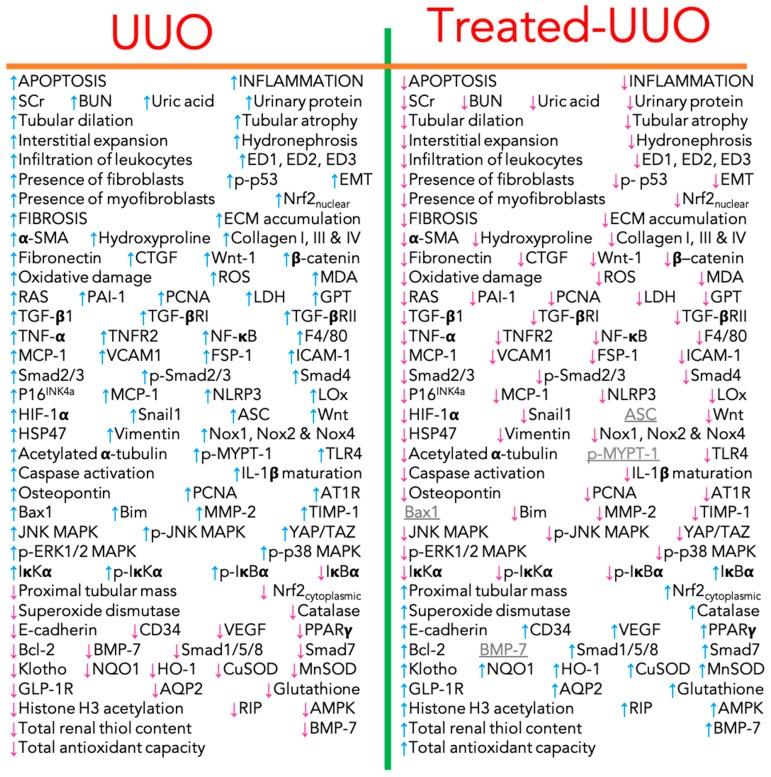
Encompassing summary of the histological lesions, protein levels or protein activities in untreated UUO and treated-UUO. The “UUO” section is the comparison between the untreated-UUO groups and the Sham-groups, whereas the “Treated-UUO” section is a comparison between the untreated-UUO groups and the treated UUO-groups. Up-arrows indicate increase, down-arrows indicate decrease and gray-underline indicates unchanged levels.

Currently, experimental treatment approximations are being sought to ameliorate or delay the onset of fibrosis in kidneys injured by ureteral obstruction. Figure 7 shows an encompassing summary of the histological lesions, protein levels or protein activities comparing the untreated-UUO-OKs/untreated-UUO-animals versus Sham-kidneys/Sham-animals and comparing the untreated-UUO-OKs/untreated-UUO-animals versus treated-UUO-OKs/treated-UUO-animals. 

Antioxidant or pharmacological treatments could have additional positive effects, especially by blocking RAS and thus, the increase in oxidative stress. Inhibiting RAS results in a decrease in the inflammatory process, the infiltration of macrophages and the levels of chemoattractants [19]. Preventing the increase in oxidative stress prevents the increase in the expression and activity of Nox and prevents the decrease of Nrf2 and of antioxidant enzymes. Therefore, the positive feedback between RAS and inflammation could be a target to delay fibrosis. Possibly, the inhibition of the NF-κB pathway results in reduced expression of angiotensin II. Reduced angiotensin II levels result in reduced protein levels of TNF-α and MCP-1 [13]. So, the use of immunosuppressive drugs has helped to mitigate renal injury in experimental models and in clinical trials of patients with CKD. 

Although the experimental treatments focus on decreased renal fibrosis in the UUO-OKs, diminishing renal fibrosis in the UUO-OKs has so far not caused death of the experimental animals, suggesting that fibrosis is a maladaptive trait, but further temporal studies are needed to avoid unwanted side effects that may appear in posterior stages. No differences in body weight are generally observed from UUO [20]. In the UUO-OKs, by compiling this review article we have noted an interesting phenomenon regarding kidney weight: in mice, UUO-OKs end up weighing less than Sham kidneys [1,30,31]; but in rats, UUO-OKs weigh more than Sham kidneys [9,10,16]. (Kidney weight is expressed as the kidney weight-to-body weight ratio).

When a treatment is administered prior to UUO, it is referred to as “preventive treatment.” Administration immediately after UUO is called “immediate administration” and administration sometime after UUO is “delayed treatment.” It was also detected that the vast majority of studies that use the UUO model focus on preventive, rather than delayed treatments. Considering that in a clinical setting, sometimes urinary obstruction cases are asymptomatic until the damage reaches advances stages, it is especially necessary to explore in more detail the effect of delayed treatments, especially after removing the obstruction, when fibrosis is already becoming established, because they would be clinically more relevant. 

Only one of the papers we revised explicitly mentioned supplementing with an antibiotic [16]; where the supplementation took place after cutting the ureter to avoid infection. If antibiotics are used, it needs to be reported, because further information is needed about the effect of antibiotic treatment on the UUO model, since it is possible that antibiotic use at any stage of the process modifies the results obtained due the mode of action of antibiotics. We must also take this opportunity to urge the scientific community to take proper care of their experimental animals, using appropriate anesthesia (the use of chloral hydrate, for instance, is controversial [66,67]) and the most suitable experimental strategy to avoid unnecessary animal suffering. 

Interestingly, a large number of the remedies that have been included in this review, especially the plant-derived compounds, have their source in traditional Chinese medicine and have proved to be effective at attenuating fibrosis. 

## 3. Conclusions

The information gathered in the present review sheds light on the treatments that have shown to be effective at attenuating the damage in an UUO model. The next step would be for this knowledge to be clinically useful and thus, further work is required. It is necessary to know what the effect of the treatment is, once the obstruction has been removed. What is the effect on an already fibrotic kidney? Does the treatment attenuate the progressive decline of renal function? To contribute to this knowledge, long-term studies are necessary, and the side effects of the treatment must be evaluated. 

## Figures and Tables

**Table 1:** Summary of technical details of UUO experiments discussed in this review.

Experimental Animal	Treatment Supplementation	Anesthesia	Ureter Ligation	Reference
Male Sprague-Dawley rats (250–280 g)	Drinking water	Isoflurane	Left, 5-0 silk	[10]
Male Sprague-Dawley rats (200–250 g)	Drinking water	Sodium pentobarbital (50 mg/kg)	Left, triple ligation, 4-0 silk,	[15]
Male Sprague-Dawley rats (200–250 g)	Intraperitoneal injection	10% Chloral hydrate (3 mL/kg)	Right, 5-0 silk, ligation in two places and cut between, penicillin	[16]
Male Sprague-Dawley rats (200–220 g)	Intraperitoneal injection	Mixture of isoflurane and oxygen, pentobarbital	Left, double ligation, 4-0 silk, cut	[4]
Male Sprague-Dawley rats (240–280 g)	Gavage in 1% carboxymethylcellulose-Na	Pentobarbital sodium (80 mg/kg)	Left, double ligation, 4-0 silk, cut	[17]
Male Sprague-Dawley rats (8 weeks old)	Gavage	Tiletamine/zolazepam (10 mg/kg)	Left, 4-0 silk, ligation at two locations and cut in between	[7]
Male Sprague-Dawley rats (180–200 g)	Gavage in 1% gum acacia	Pentobarbital sodium (50 mg/kg)	Left, 4-0 silk, triple ligation and cut	[18]
Male Sprague-Dawley rats (180–200 g)	Tube-fed	Pentobarbital	Left, 4-0 silk, ligation in two points and cut in between	[9]
Male Sprague-Dawley rats (200–250 g)	Subcutaneous implanted mini-osmotic pump and intraperitoneal injection	Isoflurane	Left, double ligation, 7-0 silk	[19]
Male Wistar rats (7 weeks old, 270–320 g)	Intraperitoneal injection	Ketamine/xylazine (70/7 mg/kg)	Left, 4-0 silk, ligation at two points and cut between	[13]
Male Wistar rats (200–220 g)	Oral administration	Ketamine/chlorpromazine (100 /0.75 mg/kg)	Left, silk	[20]
Male Wistar rats (209–247 g)	Gavage in 0.5% carboxymethylcellulose	Ketamine/xylazine hydrochloride (80/8 mg/kg)	Left, 3-4 mm long bisected PVC tube of 0.58 mm internal diameter, tube constriction with 4-0 silk (reversed UUO)	[21]
Male Wistar rats (260–305 g)	Gavage	Thiopental (100 mg/kg)	Left, 3-0 silk, double ligation	[22]
Female nu/nu mice (18–22 g, 6-8 weeks)	Intravenous injection through tail vein	Chloral hydrate 10%	Left, ligation of lower third, 4/0 suture	[14]
Male BALB/cCrSlc mice (5 weeks old)	Drinking water	Pentobarbital (50 mg/kg)	Left, ligation, 4-0 silk	[23,24]
Male BALB/c mice (6–8 weeks old, 18–22 g)	Gavage	Sodium pentobarbital (3% 10 mL/kg)	Left, double ligation, 4-0 nylon	[25]
Male C57BL/6 mice (9 weeks old)	Drinking water	Sevofrane	Left, complete ligation at the ureteropelvic junction, 4-0 silk	[26]
Male C57BL/6J mice (8 weeks old, 22–25 g)	Intraperitoneal injection	Medetomidine, midazolam, butorphanol	Left, double ligation	[27]
Male C57BL/6 mice (6–8 weeks old)	Intraperitoneal injection	3.5% Chloral hydrate (350 mg/kg)	Right, double ligation, 6-0 silk	[1]
Male C57BL/6 mice (3 months old)	Intraperitoneal injection	Pentobarbital sodium	Left, tightening at midportion, 5-0 suture	[28]
Male C57BL/6 mice (7 weeks old, 20–23 g)	Intraperitoneal injection	Ketamine/xylazine (100/10 mg/kg)	Right, double ligation, 3-0 silk	[29]
Male C57BL/6 mice (6–8 weeks old, 20–22 g)	Intraperitoneal injection	10% Chloral hydrate (3 mL/kg)	NS	[30]
Male C57BL/6 mice (6-8 weeks, 18–20 g)	Gavage	Sodium pentobarbital (1.5% 50 mg/kg)	Left, 4-0 silk, ligation at two points and cut in between	[31]
Male C57BL/6 mice (5–7 months old, 25–35 g)	Gavage	Isoflurane/oxygen	4-0 silk	[32]
Male and female C57Bl/6 mice	None	Isoflurane	Left, double ligation, 5-0 silk	[33]
Male ICR mice (18–20 g)	Injection into left kidney via ureter	Pentobarbital (75 mg/kg)	Left, complete obstruction, silk	[34]
Male ICR mice (25–30 g)	Intraperitoneal injection	Pentobarbital sodium	Left, double ligation, 4-0 silk	[35]

NS: Not Specified.

**Table 2:** Antibodies used to detect proteins by immunohistochemistry or Western Blot or gene expression measured by RT-qPCR. Arrows indicate increase or decrease in levels of UUO-OKs compared to Sham-kidneys.

Marker↓ Technique➝	Immunohistochemistry	RT-qPCR	Western Blot
18s rRNA		=[7,26]	
α-SMA	↑[8,9,10,19,26,27,29,31,38,42,51,52]	↑[1,7,15,19,25,26,27]	↑[1,7,15,17,25,26,27,29,31,32,38,50,51,52,53]
α-Tubulin			↓[53]
α-Tubulin acetylated			↑[53]
β-Actin			=[8,23,27,30,42,52,53]
β-Catenin			↑[25,54]
β-Catenin active			↑[25,54]
β-catenin_nuclear_	↑[54]		
A_1_AR, A_3_AR, A_2A_AR, A_2B_AR		↑[7]	
ACE		↓[28]	↑[25]
ACE2		↓[28]	
AGT			↑[25]
Angiotensin II	↑[13]		
ASC		↑[28,48]	↑[28,48]
AQP1, AQP2, AQP3, AQP4		↓[28]	↓[28]
p-ATM	↑[55]		
AT_1_R		↑[28]	↑[8,25]
Bax			↑[8]
Bcl-2			↓[8]
Bim			↑[8]
BMP-7	↓[35]	↓[35]	↓[35]
Caspase-1		↑[28,48]	↑[28]
Caspase-1 cleaved			↑[48]
pro-Caspase-1			↑[48]
Caspase-3 cleaved			↑[1,53]
Caspase-8		↑[22]	
Caspase-9			↑[53]
Catalase			↓[8]
CD3	↑[48]		
CD34	↓[14]		
Collagen I	↑[14,17,27,29,42,49,51]	↑[7,15,26,27,38]	↑[7,14,15,17,25,31,32,51,52]
Collagen III	↑[17,27]	↑[18,26,27]	↑[17,32]
Collagen IV	↑[56]	↑[1,23,24]	↑[1,31]
CTGF	↑[20,57]		↑[33,57]
CuSOD			↓[8]
E-cadherin	↓[14,32,38,42,51,52]	↑[7]	↓[7,14,25,38,51,52,54] =[53]
ED1 (CD68)	↑[7,9,10,27,48]		↑[53]
ED2 (CD163)	↑[10]		
ED3 (CD169)	↑[10]		
EGFR	↑[57]		
p-EGFR			↑[57]
ERK1/2 MAPK			↑[1,23,38] = [51]
p-ERK1/2-MAPK			↑[1,7,23,24,38,51]
F4/80	↑[8,24,26,29,32]	↑[26]	
Fibronectin	↑[27,32,42,49,51]	↑[1,7,15,26,27,38]	↑[1,7,15,25,27,29,32,49,51,52,53]
FSP-1 (S100A4)	↑[27,29,35]		↑[25,35]
GAPDH (G3PDH)		=[1,10,15,17,23,24,32,51]	=[17,26,27,29,31,38,49]
GLP-1R	↓[38]		
GSTa2		=[8]	
GSTm3		↓[8]	
HIF-1[α]	↑[14]		↑[14]
Histone H3			=[29]
Histone H3 acetylated			=[29]
HO-1		=[8]	↓[8] ↑[30]
HSP47	↑[23]	↑[24]	
IκBα			↓[17,30]
p-IκBα			↑[17,30]
IκKα			↑[17]
p-IκKα			↑[17]
ICAM-1	↑[29]	↑[18]	↑[29]
IL-1[β]	↑[17]	↑[[28,48]	↑[17,28]
Cleaved-IL-1β			↑[48]
pro-IL-1β			↑[48]
IL-6		↑[28]	
IL-17		↑[15]	↑[15]
IL-18		↑[28]	
JNK-MAPK			↑[1,51] =[23]
p-JNK-MAPK			↑[1,7,23,24,51]
Keap1			↑[8]
Ki-67	↑[14,29]		
Klotho		↓[20]	↓[31]
LRP5 LRP6			↑[54]
p-LRP5 p-LRP6			↑[54]
Lysyl oxidase (LOx)		↑[7]	
MCP-1	↑[9,13,14,29]	↑[7,18,19,26,28]	↑[14]
MMP2		↑[27]	↑[17,27,54]
MMP7		↑[25]	↑[54]
MMP9		↑[27]	↑[27]
MnSOD			↓[8]
p-MYPT-1			↑[26]
NF-κB			↑[17,30]
p-NF-κB			↑[17]
NLRP3		↑[28,48]	↑[48]
Nox1, Nox2, Nox4			↑[8]
NQO1		↓[8]	↓[8]
Nrf2_cytoplasmic_			↓[8] =[30]
Nrf2_nuclear_			=[8] ↑[30]
OPN		↑[19]	
P16^INK4a^	↑[35]	↑[35]	↑[35]
p22^Phox^			↑[55,57]
p38-MAPK			↑[1] = [51]
p-p38-MAPK			↑[1,51]
p53	↑[57,58]		↑[58]
p-p53			↑[55,57]
PAI-1	↑[57]	↑[18]	↑[25,33,52,57]
PCNA	↑[14]	↑[15]	↑[15]
PPAR-𝝲			↑[49]
PRR		↑[28]	
Renin		↑[19,28]	↑[25]
Renin Receptor		↑[28]	
ROCK1 and ROCK2		↑[26]	
RIP		↓[22]	
Smad1/5/8			↓[35]
Smad2		↑[17,35]	↑[17,25,27] = [51]
p-Smad2			↑[17,25,29,51]
Smad3		↑[17]	↑[17,25,27,51] = [38,52]
p-Smad3			↑[7,17,25,26,29,33,38,51,52,55,57]
Smad2/3	↑[31,35]		↑[29,35,49]
p-Smad2/3			↑[31,57]
Smad4			↑[25,27,31,51]
Smad7			↓[25,29,31]
Smad8		↓[35]	
Snail1	↑[38]		↑[25,54]
p-Src			↑[59]
STAT3			=[52]
p-STAT3			↑[52]
TAZ	↑[33,56]		↑[33]
TIMP-1			↑[17]
TGF-β_1_	↑ [9,14,31,35,38]	↑[1,7,10,17,19,24,26,27,28,35,38]	↑[14,17,31,32,35,51,53]
TGF-βRI	↑[38]	↑[38]	↑[27,31]
TGF-βRII			↓[27] ↑[31]
TLR4			↑[17]
TNF-α	↑[17]	↑[28]	↑[17,32]
TNFR1		=[22]	
TNFR2		↑[22]	
TRAF2		↓[22]	
Twist			↑[25,54]
V2R		↓[28]	
VCAM-1			↑[32]
VEGF	↓[14]		↓[14]
Vimentin	↑[42]		↑[29]
Wnt1		↓[54]	↑[25]
Wnt2		↑[54]	
Wnt3	↑[54]	↑[54]	↑[54]
Wnt4	↑[54]	↑[54]	↑[54]
YAP	↑[56]		↑[33]

**Table 3:** Summary of the treatments to ameliorate renal fibrosis discussed in this review.

Treatment	Amount Supplied	Time of Supplementation	Days until Euthanasia	Reference
Aliskiren	20 mg/kg/day (3 or 7 days)	Immediately before UUO	3, 7	[28]
Aliskiren and MZR	20 mg/kg/day and 10 mg/kg/day	One day after and daily	14	[19]
Alpha-lipoic acid	60 mg/kg/day	Two days before and daily	7	[4]
AKBA	10, 20, 40 mg/kg/day	Immediately after and daily	14	[31]
Amlodipine	6.7 mg/kg/day	Immediately after	7	[24]
Amygdalin	3, 5 mg/kg/day	Immediately after and daily	7, 14, 21	[39]
Applephenon	0.05, 0.1, 0.15% where 0.1% is 40 mg/kg/day	One day after and daily	7, 14, 21	[9]
ASPA	10, 20, 40 mg/kg/day	Immediately after and daily	14	[16]
AS-IV	20 mg/kg/day	Immediately after and daily	7, 14	[1]
BAIBA	150 mg/kg/day	Immediately after and daily	14	[15]
Colchicine	30, 60, 100 µg/kg/day	Immediately after and daily	7	[53]
Cryptotanshinone	50 mg/kg/day	Seven days before and daily	7	[51]
Curcumin	50, 100 mg/kg/day	One day after and daily	14	[49]
Curcumin	200 mg/kg/day	Five days before and daily	3	[21]
Curcumin	200, 800 mg/kg/day	Seven days before and daily	7	[18]
DIM	100 mg/kg/day	Four weeks before and daily	7	[42]
Empagliflozin	10 mg/kg/day	One week before and daily	14	[20]
Empagliflozin	10 mg/kg/day	Immediately after	14	[20]
Empagliflozin	10 mg/kg/day	One week after and daily	21	[20]
EGCG	50 mg/kg/day	Immediately after and daily	14	[30]
Erythropoietin	1000 U/kg/day	One day before and every other day	3, 7, 14	[35]
Fasudil	1 g/L	Two days before and daily	3, 7, 14	[26]
Fimasartan	3 mg/kg/day	Immediately after and daily	7	[8]
Fluorofenidone	500 mg/kg/day	One day before and daily	3, 7	[48]
hAFSCs	3.5 × 10^5^ cells	Immediately after	1, 3, 7, 14	[14]
HSP47 siRNA	50 µg/mouse	Immediately before	0, 7, 14	[34]
Liraglutide	600 µg/kg/day	Immediately after and daily	7	[38]
LJ-1888	1, 10 mg/kg	Five days before and daily	5	[7]
LJ-1888	1, 10 mg/kg	Three days after	10	[7]
Meloxicam	1 mg/kg/day	Immediately after and daily	7	[23]
Metformin	200 mg/kg/day	One day before	7, 14	[32]
Pomolic acid	0.4 mg/kg/day	Immediately after and two days after	7	[52]
Poricoic acids	5, 10, 20, 40 mg/kg/day	Immediately after and daily	7	[25]
PR-619	100 µg per day	Immediately after	7	[27]
Telbivudine	1, 1.5, 2 g/kg/day	From day two and daily	36	[17]
Thymoquinone	10 mg/kg	Three days before and daily	14	[13]
Valproic acid	300 mg/kg/day	Five days before and daily	14	[29]
Verteporfin	100 mg/kg	Every other day after	7, 14	[56]
Verteporfin	100 mg/kg	Seven days after	7, 14	[56]

## References

[1] Xu W., Shao X., Tian L., Gu L., Zhang M., Wang Q., Wu B., Wang L., Yao J., Xu X. (2014). Astragaloside IV Ameliorates Renal Fibrosis via the Inhibition of Mitogen-Activated Protein Kinases and Antiapoptosis In Vivo and In Vitro. J. Pharmacol. Exp. Ther..

[2] Chevalier R.L., Forbes M.S., Thornhill B.A. (2009). Ureteral obstruction as a model of renal interstitial fibrosis and obstructive nephropathy. Kidney Int..

[3] Chevalier R.L., Thornhill B.A., Forbes M.S., Kiley S.C. (2010). Mechanisms of renal injury and progression of renal disease in congenital obstructive nephropathy. Pediatr. Nephrol..

[4] Wongmekiat O., Leelarungrayub D., Thamprasert K. (2013). Alpha-Lipoic Acid Attenuates Renal Injury in Rats with Obstructive Nephropathy. Biomed Res. Int..

[5] Ucero A.C., Benito-Martin A., Izquierdo M.C., Sanchez-Niño M.D., Sanz A.B., Ramos A.M., Berzal S., Ruiz-Ortega M., Egido J., Ortiz A. (2014). Unilateral ureteral obstruction: beyond obstruction. Int. Urol. Nephrol..

[6] Zhang J., Xing Z.-Y., Zha T., Tian X.-J., Du Y.-N., Chen J., Xing W. (2018). Longitudinal assessment of rabbit renal fibrosis induced by unilateral ureteral obstruction using two-dimensional susceptibility weighted imaging. J. Magn. Reson. Imaging.

[7] Lee J., Hwang I., Lee J.H., Lee H.W., Jeong L.-S., Ha H. (2013). The Selective A 3 AR Antagonist LJ-1888 Ameliorates UUO-Induced Tubulointerstitial Fibrosis. Am. J. Pathol..

[8] Kim S., Kim S.J., Yoon H.E., Chung S., Choi B.S., Park C.W., Shin S.J. (2015). Fimasartan, a Novel Angiotensin-Receptor Blocker, Protects against Renal Inflammation and Fibrosis in Mice with Unilateral Ureteral Obstruction: the Possible Role of Nrf2. Int. J. Med. Sci..

[9] Lee W.-C., Jao H.-Y., Hsu J.-D., Lee Y.-R., Wu M.-J., Kao Y.-L., Lee H.-J. (2014). Apple polyphenols reduce inflammation response of the kidneys in unilateral ureteral obstruction rats. J. Funct. Foods.

[10] Kido T., Tsunoda M., Sugaya C., Hano H., Yanagisawa H. (2017). Fluoride potentiates tubulointerstitial nephropathy caused by unilateral ureteral obstruction. Toxicology.

[11] Chaabane W., Praddaude F., Buleon M., Jaafar A., Vallet M., Rischmann P., Galarreta C.I., Chevalier R.L., Tack I. (2013). Renal functional decline and glomerulotubular injury are arrested but not restored by release of unilateral ureteral obstruction (UUO). Am. J. Physiol. Physiol..

[12] Yanagisawa H., Nodera M., Wada O. (1998). Zinc deficiency aggravates tubulointerstitial nephropathy caused by ureteral obstruction. Biol. Trace Elem. Res..

[13] Hosseinian S., Rad A.K., Bideskan A.E., Soukhtanloo M., Sadeghnia H., Shafei M.N., Motejadded F., Mohebbati R., Shahraki S., Beheshti F. (2017). Thymoquinone ameliorates renal damage in unilateral ureteral obstruction in rats. Pharmacol. Reports.

[14] Sun D., Bu L., Liu C., Yin Z., Zhou X., Li X., Xiao A. (2013). Therapeutic Effects of Human Amniotic Fluid-Derived Stem Cells on Renal Interstitial Fibrosis in a Murine Model of Unilateral Ureteral Obstruction. PLoS ONE.

[15] Wang H., Qian J., Zhao X., Xing C., Sun B. (2017). β-Aminoisobutyric acid ameliorates the renal fibrosis in mouse obstructed kidneys via inhibition of renal fibroblast activation and fibrosis. J. Pharmacol. Sci..

[16] Xianyuan L., Wei Z., Yaqian D., Dan Z., Xueli T., Zhanglu D., Guanyi L., Lan T., Menghua L. (2019). Anti-renal fibrosis effect of asperulosidic acid via TGF-β1/smad2/smad3 and NF-κB signaling pathways in a rat model of unilateral ureteral obstruction. Phytomedicine.

[17] Chen J., Li D. (2018). Telbivudine attenuates UUO-induced renal fibrosis via TGF-β/Smad and NF-κB signaling. Int. Immunopharmacol..

[18] Kuwabara N., Tamada S., Iwai T., Teramoto K., Kaneda N., Yukimura T., Nakatani T., Miura K. (2006). Attenuation of renal fibrosis by curcumin in rat obstructive nephropathy. Urology.

[19] Sakuraya K., Endo A., Someya T., Hirano D., Murano Y., Fujinaga S., Ohtomo Y., Shimizu T. (2014). The Synergistic Effect of Mizoribine and a Direct Renin Inhibitor, Aliskiren, on Unilateral Ureteral Obstruction Induced Renal Fibrosis in Rats. J. Urol..

[20] Abbas N.A.T., El. Salem A., Awad M.M. (2018). Empagliflozin, SGLT2 inhibitor, attenuates renal fibrosis in rats exposed to unilateral ureteric obstruction: potential role of klotho expression. Naunyn. Schmiedebergs. Arch. Pharmacol..

[21] Hammad F.T., Lubbad L. (2011). Does Curcumin Protect against Renal Dysfunction following Reversible Unilateral Ureteric Obstruction in the Rat?. Eur. Surg. Res..

[22] Hashem R.M., Mohamed R.H., Abo-El-matty D.M. (2016). Effect of curcumin on TNFR2 and TRAF2 in unilateral ureteral obstruction in rats. Nutrition.

[23] Honma S., Shinohara M., Takahashi N., Nakamura K., Hamano S., Mitazaki S., Abe S., Yoshida M. (2014). Effect of cyclooxygenase (COX)-2 inhibition on mouse renal interstitial fibrosis. Eur. J. Pharmacol..

[24] Honma S., Nakamura K., Shinohara M., Mitazaki S., Abe S., Yoshida M. (2016). Effect of amlodipine on mouse renal interstitial fibrosis. Eur. J. Pharmacol..

[25] Wang M., Chen D.-Q., Chen L., Cao G., Zhao H., Liu D., Vaziri N.D., Guo Y., Zhao Y.-Y. (2018). Novel inhibitors of the cellular renin-angiotensin system components, poricoic acids, target Smad3 phosphorylation and Wnt/β-catenin pathway against renal fibrosis. Br. J. Pharmacol..

[26] Baba I., Egi Y., Utsumi H., Kakimoto T., Suzuki K. (2015). Inhibitory effects of fasudil on renal interstitial fibrosis induced by unilateral ureteral obstruction. Mol. Med. Rep..

[27] Soji K., Doi S., Nakashima A., Sasaki K., Doi T., Masaki T. (2018). Deubiquitinase inhibitor PR-619 reduces Smad4 expression and suppresses renal fibrosis in mice with unilateral ureteral obstruction. PLoS ONE.

[28] Wang W., Luo R., Lin Y., Wang F., Zheng P., Levi M., Yang T., Li C. (2015). Aliskiren restores renal AQP2 expression during unilateral ureteral obstruction by inhibiting the inflammasome. Am. J. Physiol. Physiol..

[29] Nguyen-Thanh T., Kim D., Lee S., Kim W., Park S., Kang K. (2017). Inhibition of histone deacetylase 1 ameliorates renal tubulointerstitial fibrosis via modulation of inflammation and extracellular matrix gene transcription in mice. Int. J. Mol. Med..

[30] Wang Y., Wang B., Du F., Su X., Sun G., Zhou G., Bian X., Liu N. (2015). Epigallocatechin-3-Gallate Attenuates Oxidative Stress and Inflammation in Obstructive Nephropathy via NF-κB and Nrf2/HO-1 Signalling Pathway Regulation. Basic Clin. Pharmacol. Toxicol..

[31] Liu M., Liu T., Shang P., Zhang Y., Liu L., Liu T., Sun S. (2018). Acetyl-11-keto-β-boswellic acid ameliorates renal interstitial fibrosis via Klotho/TGF-β/Smad signalling pathway. J. Cell. Mol. Med..

[32] Cavaglieri R.C., Day R.T., Feliers D., Abboud H.E. (2015). Metformin prevents renal interstitial fibrosis in mice with unilateral ureteral obstruction. Mol. Cell. Endocrinol..

[33] Anorga S., Overstreet J.M., Falke L.L., Tang J., Goldschmeding R.G., Higgins P.J., Samarakoon R. (2018). Deregulation of Hippo–TAZ pathway during renal injury confers a fibrotic maladaptive phenotype. FASEB J..

[34] Xia Z., Abe K., Furusu A., Miyazaki M., Obata Y., Tabata Y., Koji T., Kohno S. (2008). Suppression of Renal Tubulointerstitial Fibrosis by Small Interfering RNA Targeting Heat Shock Protein 47. Am. J. Nephrol..

[35] Tasanarong A., Kongkham S., Khositseth S. (2013). Dual Inhibiting Senescence and Epithelial-to-Mesenchymal Transition by Erythropoietin Preserve Tubular Epithelial Cell Regeneration and Ameliorate Renal Fibrosis in Unilateral Ureteral Obstruction. Biomed Res. Int..

[36] Bolati D., Shimizu H., Yisireyili M., Nishijima F., Niwa T. (2013). Indoxyl sulfate, a uremic toxin, downregulates renal expression of Nrf2 through activation of NF-κB. BMC Nephrol..

[37] Sedeek M., Nasrallah R., Touyz R.M., Hebert R.L. (2013). NADPH Oxidases, Reactive Oxygen Species, and the Kidney: Friend and Foe. J. Am. Soc. Nephrol..

[38] Li Y.-K., Ma D.-X., Wang Z.-M., Hu X.-F., Li S.-L., Tian H.-Z., Wang M.-J., Shu Y.-W., Yang J. (2018). The glucagon-like peptide-1 (GLP-1) analog liraglutide attenuates renal fibrosis. Pharmacol. Res..

[39] Guo J., Wu W., Sheng M., Yang S., Tan J. (2013). Amygdalin inhibits renal fibrosis in chronic kidney disease. Mol. Med. Rep..

[40] LeBleu V.S., Taduri G., O’Connell J., Teng Y., Cooke V.G., Woda C., Sugimoto H., Kalluri R. (2013). Origin and function of myofibroblasts in kidney fibrosis. Nat. Med..

[41] Farris A.B., Alpers C.E. (2014). What is the best way to measure renal fibrosis?: A pathologist’s perspective. Kidney Int. Suppl..

[42] Xia Z.-E., Xi J.-L., Shi L. (2018). 3,3′-Diindolylmethane ameliorates renal fibrosis through the inhibition of renal fibroblast activation in vivo and in vitro. Ren. Fail..

[43] Kalluri R., Weinberg R.A. (2009). The basics of epithelial-mesenchymal transition. J. Clin. Invest..

[44] Sato M., Muragaki Y., Saika S., Roberts A.B., Ooshima A. (2003). Targeted disruption of TGF-β1/Smad3 signaling protects against renal tubulointerstitial fibrosis induced by unilateral ureteral obstruction. J. Clin. Invest..

[45] Chevalier R.L., Kim A., Thornhill B.A., Wolstenholme J.T. (1999). Recovery following relief of unilateral ureteral obstruction in the neonatal rat. Kidney Int..

[46] Ito K., Chen J., El Chaar M., Stern J.M., Seshan S.V., Khodadadian J.J., Richardson I., Hyman M.J., Vaughan E.D., Poppas D.P. (2004). Renal damage progresses despite improvement of renal function after relief of unilateral ureteral obstruction in adult rats. Am. J. Physiol. Physiol..

[47] Demirbilek S., Emre M.H., Aydın E.N., Edali M.N., Aksoy R.T., Akın M., Gürünlüoğlu K., Tas E., Ay S., Yilmaz Z. (2007). Sulfasalazine reduces inflammatory renal injury in unilateral ureteral obstruction. Pediatr. Nephrol..

[48] Zheng L., Zhang J., Yuan X., Tang J., Qiu S., Peng Z., Yuan Q., Xie Y., Mei W., Tang Y. (2018). Fluorofenidone attenuates interleukin-1β production by interacting with NLRP3 inflammasome in unilateral ureteral obstruction. Nephrology.

[49] Zhou X., Zhang J., Xu C., Wang W. (2014). Curcumin Ameliorates Renal Fibrosis by Inhibiting Local Fibroblast Proliferation and Extracellular Matrix Deposition. J. Pharmacol. Sci..

[50] Zhang Z.-H., He J.-Q., Qin W.-W., Zhao Y.-Y., Tan N.-H. (2018). Biomarkers of obstructive nephropathy using a metabolomics approach in rat. Chem. Biol. Interact..

[51] Wang W., Zhou P.-H., Hu W., Xu C.-G., Zhou X.-J., Liang C.-Z., Zhang J. (2018). Cryptotanshinone hinders renal fibrosis and epithelial transdifferentiation in obstructive nephropathy by inhibiting TGF-B1;1/Smad3/integrin B1 signal. Oncotarget.

[52] Park J.-H., Jang K., An H., Kim J.-Y., Gwon M.-G., Gu H., Park B., Park K.-K. (2018). Pomolic Acid Ameliorates Fibroblast Activation and Renal Interstitial Fibrosis through Inhibition of SMAD-STAT Signaling Pathways. Molecules.

[53] Kim S., Jung E.S., Lee J., Heo N.J., Na K.Y., Han J.S. (2018). Effects of colchicine on renal fibrosis and apoptosis in obstructed kidneys. Korean J. Intern. Med..

[54] Wang L., Chi Y.-F., Yuan Z.-T., Zhou W.-C., Yin P.-H., Zhang X.-M., Peng W. (2014). Astragaloside IV Inhibits the Up-Regulation of Wnt/β-Catenin Signaling in Rats with Unilateral Ureteral Obstruction. Cell. Physiol. Biochem..

[55] Overstreet J.M., Samarakoon R., Cardona-Grau D., Goldschmeding R., Higgins P.J. (2015). Tumor suppressor ataxia telangiectasia mutated functions downstream of TGF-β 1 in orchestrating profibrotic responses. FASEB J..

[56] Szeto S.G., Narimatsu M., Lu M., He X., Sidiqi A.M., Tolosa M.F., Chan L., De Freitas K., Bialik J.F., Majumder S. (2016). YAP/TAZ Are Mechanoregulators of TGF-β -Smad Signaling and Renal Fibrogenesis. J. Am. Soc. Nephrol..

[57] Samarakoon R., Dobberfuhl A.D., Cooley C., Overstreet J.M., Patel S., Goldschmeding R., Meldrum K.K., Higgins P.J. (2013). Induction of renal fibrotic genes by TGF-β1 requires EGFR activation, p53 and reactive oxygen species. Cell. Signal..

[58] Overstreet J.M., Samarakoon R., Meldrum K.K., Higgins P.J. (2014). Redox control of p53 in the transcriptional regulation of TGF-β1 target genes through SMAD cooperativity. Cell. Signal..

[59] Yan Y., Ma L., Zhou X., Ponnusamy M., Tang J., Zhuang M.A., Tolbert E., Bayliss G., Bai J., Zhuang S. (2016). Src inhibition blocks renal interstitial fibroblast activation and ameliorates renal fibrosis. Kidney Int..

[60] National Center for Biotechnology Information, U.S. National Library of Medicine, N.I. of H. PubChem. https://pubchem.ncbi.nlm.nih.gov.

[61] Blaheta R.A., Nelson K., Haferkamp A., Juengel E. (2016). Amygdalin, quackery or cure?. Phytomedicine.

[62] Sun X., Liu Y., Li C., Wang X., Zhu R., Liu C., Liu H., Wang L., Ma R., Fu M. (2017). Recent Advances of Curcumin in the Prevention and Treatment of Renal Fibrosis. Biomed Res. Int..

[63] Yang R., Xu X., Li H., Chen J., Xiang X., Dong Z., Zhang D. (2017). p53 induces miR199a-3p to suppress SOCS7 for STAT3 activation and renal fibrosis in UUO. Sci. Rep..

[64] Conductier G., Blondeau N., Guyon A., Nahon J.-L., Rovère C. (2010). The role of monocyte chemoattractant protein MCP1/CCL2 in neuroinflammatory diseases. J. Neuroimmunol..

[65] Wolf G., Jocks T., Zahner G., Panzer U., Stahl R.A.K. (2002). Existence of a regulatory loop between MCP-1 and TGF-β in glomerular immune injury. Am. J. Physiol. Physiol..

[66] Baxter M.G., Murphy K.L., Taylor P.M., Wolfensohn S.E. (2009). Chloral Hydrate Is Not Acceptable for Anesthesia or Euthanasia of Small Animals. Anesthesiology.

[67] Ren Y., Zhang F.-J., Xue Q.-S., Zhao X., Yu B.-W. (2009). Chloral Hydrate Is Not Acceptable for Anesthesia or Euthanasia of Small Animals. Anesthesiology.

